# Multi ‘Omics Analysis of Intestinal Tissue in Ankylosing Spondylitis Identifies Alterations in the Tryptophan Metabolism Pathway

**DOI:** 10.3389/fimmu.2021.587119

**Published:** 2021-03-03

**Authors:** Adam J. Berlinberg, Emilie H. Regner, Andrew Stahly, Ana Brar, Julie A. Reisz, Mark E. Gerich, Blair P. Fennimore, Frank I. Scott, Alison E. Freeman, Kristine A. Kuhn

**Affiliations:** ^1^ Division of Rheumatology, Department of Medicine, University of Colorado, Aurora, CO, United States; ^2^ Division of Gastroenterology and Hepatology, Department of Medicine, University of Colorado, Aurora, CO, United States; ^3^ Department of Medicine, University of Colorado, Aurora, CO, United States; ^4^ Department of Biochemistry and Molecular Genetics, University of Colorado, Aurora, CO, United States

**Keywords:** ankylosing spondylitis, spondyloarthritis, metabolomics, tryptophan, metagenomics, indole, microbiome

## Abstract

Intestinal microbial dysbiosis, intestinal inflammation, and Th17 immunity are all linked to the pathophysiology of spondyloarthritis (SpA); however, the mechanisms linking them remain unknown. One potential hypothesis suggests that the dysbiotic gut microbiome as a whole produces metabolites that influence human immune cells. To identify potential disease-relevant, microbiome-produced metabolites, we performed metabolomics screening and shotgun metagenomics on paired colon biopsies and fecal samples, respectively, from subjects with axial SpA (axSpA, N=21), Crohn’s disease (CD, N=27), and Crohn’s-axSpA overlap (CD-axSpA, N=12), as well as controls (HC, N=24). Using LC-MS based metabolomics of 4 non-inflamed pinch biopsies of the distal colon from subjects, we identified significant alterations in tryptophan pathway metabolites, including an expansion of indole-3-acetate (IAA) in axSpA and CD-axSpA compared to HC and CD and indole-3-acetaldehyde (I3Ald) in axSpA and CD-axSpA but not CD compared to HC, suggesting possible specificity to the development of axSpA. We then performed shotgun metagenomics of fecal samples to characterize gut microbial dysbiosis across these disease states. In spite of no significant differences in alpha-diversity among the 4 groups, our results confirmed differences in gene abundances of numerous enzymes involved in tryptophan metabolism. Specifically, gene abundance of indolepyruvate decarboxylase, which generates IAA and I3Ald, was significantly elevated in individuals with axSpA while gene abundances in HC demonstrated a propensity towards tryptophan synthesis. Such genetic changes were not observed in CD, again suggesting disease specificity for axSpA. Given the emerging role of tryptophan and its metabolites in immune function, altogether these data indicate that tryptophan metabolism into I3Ald and then IAA is one mechanism by which the gut microbiome potentially influences the development of axSpA.

## Introduction

Ankylosing spondylitis is a form of axial spondyloarthritis (axSpA) resulting in inflammation of the axial spine, peripheral joints, and entheses (1). The clinical overlap between axSpA and bowel inflammation has long suggested an interaction between the gut and joint in its pathogenesis (2), and the condition of reactive arthritis caused by intestinal pathogens indicates that intestinal bacteria may trigger some forms of disease. Intestinal microbiome studies in humans with axSpA have shown significant ecological alterations (dysbiosis) in bacterial taxa such as *Ruminococcus gnavus, Dialister*, and *Akkermansia muciniphila* as compared to healthy controls (3–7). Few studies have demonstrated consensus taxa, though, which may be due to geographic and other study-specific influences, yet raises the question of how disparate bacterial species can contribute to pathogenesis of disease.

In the HLA-B27/β2m transgenic rat model that develops spontaneous SpA and bowel inflammation, transcriptional changes in IL-17, IL-23, and TNF, key cytokines in the pathophysiology of SpA (8), in the intestinal tissues are associated with metabolic changes as well as microbial changes that are found in human SpA (9). This finding suggests that dysbiosis can influence pathogenic immunity of SpA. Additional work utilizing rats with different genetic backgrounds indicates that community functions rather than specific taxa may be key to disease development. Using two susceptible rat strains for the development of intestinal inflammation in the setting of B27, dysbiosis was vastly different between the two strains, lacking a common taxonomic profile to associate with disease and specific cytokine production. However, when metabolic pathways were imputed from the dysbiotic ecosystem, common features of vitamin synthesis and short-chain fatty acid synthesis emerged (10). Indeed, murine studies have addressed the concept of intestinal bacterial metabolites such as riboflavin and short chain fatty acids like butyrate influencing immune cells, particularly Th17 cells (8, 11–14). In the B27 rat model, both intestinal bacteria and short and medium-chain fatty acid metabolites are likely important for establishing Th17-mediated inflammation (15, 16). Thus, community function leading to metabolic alterations that affect mucosal immunity may be more relevant than specific taxa in influencing pathophysiology.

Despite the described dysbiosis in axSpA, a number of knowledge gaps remain: First, what are the microbial community functions in human axSpA? As described above, there are metabolic changes in rats with B27-related SpA. Furthermore, alterations in a number of bacterially-generated metabolites have been found to be altered in a similar SpA disease in humans, psoriatic arthritis (PsA) (3, 17, 18). Yet, such analyses have not been performed in human axSpA. Second, microbiome studies in axSpA to date have been inclusive of subjects with intestinal inflammation, which is present in nearly 50% of axSpA patients on a histologic level (19). How this serves as a confounding factor remains unclear. In this study, we hypothesized that the intestinal microbiome associates with an altered metabolomic profile in axSpA distinct from controls and confounding by intestinal inflammation. Within this work, we utilize an unbiased metabolomics approach followed by metagenomics methods to investigate alterations in metabolic byproducts of the bacterial communities and how these can relate to the gut microbiome using a pure axSpA cohort compared to those with intestinal inflammation.

## Methods

### Subject Recruitment

Utilizing a case-control format, patient and control study subjects were recruited at the University of Colorado Hospital between November 2017 and November 2018. Subjects were identified from the endoscopy schedule if undergoing a routine colonoscopy as part of their clinical care or recruited to undergo an elective flexible sigmoidoscopy. Recruited healthy controls (HC) (n=24) were undergoing colonoscopy for routine cancer screening or a change in bowel habits. AxSpA cases (n=21) underwent elective flexible sigmoidoscopy for the purpose of this study or were undergoing colonoscopy due to changes in bowel habits (n=2), and were only included as cases when IBD was excluded macroscopically and histologically. Subjects with CD (n=27) were undergoing colonoscopies for disease activity assessment and colon cancer/dysplasia screening. Only patients without endoscopic or histologic evidence of dysplasia were recruited into the study. Additionally, patients with both CD and AxSpA (CD-axSpA) (n=12) were recruited and similarly underwent either elective flexible sigmoidoscopy for the purpose of this study (n=8) or standard of care colonoscopy (n=4). Subjects with CD were eligible if they had biopsy-proven CD with terminal ileum involvement during the history of their disease. Subjects recruited as axSpA cases fulfilled the 2009 Assessment of SpondyloArthritis International Society (ASAS) criteria for axSpA (20), including evidence of axial disease by either MRI or radiographs. Individuals with CD-axSpA met study criteria for both CD and axSpA. Exclusion criteria for all groups included: presence of bowel disease, pregnancy, use of antibiotics in the two weeks prior to study entry, cancer or cancer history, inability to stop aspirin or non-steroidal anti-inflammatory drugs seven days before and after endoscopy, use of anticoagulation, HIV, and *Clostridium difficile* infection within the past 3 months.

At the time of endoscopy, subjects completed questionnaires regarding demographic information and disease activity through the Bath Ankylosing Spondylitis Disease Activity Index (BASDAI) and Harvey Bradshaw Index. These data are presented in **Supplemental Table 1**. Prior to colonoscopy, a rectal swab was also performed on each subject. Swabs (BD FecalSwab) were inserted 3 cm past the anal verge and rotated against the lateral colon wall a minimum of three times. Swabs were then placed immediately on ice and frozen at −80°C until further use. Thirty pinch biopsies from uninflamed rectosigmoid colon were taken during endoscopy, placed into RPMI 1640 (Gibco) on ice, and stored cryogenically in recovery freezing media (Gibco) until further analysis.

This study was conducted according to the principles within the Declaration of Helsinki. All study procedures were approved by the Colorado Multiple Institutional Review Board. All subjects provided written informed consent.

### Metabolomics

Following initial colonic pinch biopsies, four previously frozen dry samples were randomly chosen and analyzed *via* ultra-high pressure liquid chromatography-mass spectrometry-based high throughput metabolomics at the University of Colorado School of Medicine Metabolomics Core. Frozen tissue samples were weighed to 15 mg tissue per mL extraction buffer then extracted at 20 mg/mL using ice-cold 5:3:2 methanol:acetonitrile:water (v/v/v) in the presence of glass beads at 4°C. Samples were homogenized using a bead beater for 5 min then vortexed 30 min at 4°C, spun down for 10 min at 18,000 rcf and 4°C, and the supernatants analyzed on a Thermo Vanquish UHPLC coupled to a Thermo Q Exactive mass spectrometer. Metabolites were separated on a 5 min C18 gradient with positive and negative (separate runs) using electrospray ionization. Detailed data acquisition parameters and chromatographic conditions are described in a recent methods paper (21, 22). Quality control was assessed using technical replicates injected every 10 runs. Resulting.raw files were converted to.mzXML format using RawConverter then metabolites assigned and peak areas integrated using Maven (Princeton University) in conjunction with the KEGG database and an in-house standard library of >600 compounds. The targeted data analysis focused on metabolites involved in central carbon and nitrogen metabolism and yielded measurements of 184 metabolites. No *post hoc* normalization was performed; data is available upon request. Samples were normalized relative to each other based upon the same initial starting weight of tissue.

### Microbial DNA Extraction, Library Prep, and Metagenomics Sequencing

DNA was isolated from previously described rectal swabs using the Qiagen AllPrep Power Fecal DNA/RNA kit. Standard protocol was followed per kit instructions. Quality control was performed using a Thermo Scientific NanoDrop 2000 spectrophotometer ensuring 260/280 nm light ratios >1.7 for all samples. Libraries were then constructed using the NEB Next Ultra II FS DNA Library Prep Kit in a paired end fashion with 2x150 base pair paired end reads. One hundred fifty nanograms of DNA was utilized for each sample in creating libraries. Libraries underwent quality control *via* tape station prior to multiplexing at a concentration of 4 nM, and sequencing was performed on an Illumina NovaSeq6000 platform (San Diego, CA, USA) at the University of Colorado Genomics core with >6 Gb data output per sample.

### Data Processing and Taxonomic Classification

Manual inspection of sequenced reads was performed utilizing FastQC v0.11.9 for all samples. Paired end reads were then concatenated and quality control conducted with Kneaddata 0.7.5 (http://huttenhower.sph.harvard.edu/kneaddata), utilizing Trimmomatic v0.39 (23) and Bowtie2 v2.3.5 (24) to remove unwanted human genome reads and low quality sequences. The processed reads were then entered into the HUMAnN 2.0 pipeline (25), utilizing MetaPhlAn v2.0 (26), which does not account for paired-end relationships, with gene profiling abundance performed using the UniRef90 full universal database. Output data in reads per kilobase was then converted to copies per million prior to downstream application. Taxonomic profiling, alpha diversity, and beta diversity were performed in MicrobiomeAnalyst (27, 28). EdgeR was used at standard settings to perform statistical analysis of previously obtained taxonomic profiling.

### Metagenome Analysis

Functional output from HUMAnN 2.0 regarding gene families from all samples was merged in a pairwise manner using the command: humann2_join_tables. Output from HUMAnN 2.0 in reads per kilobase was then converted to copies per million prior to downstream application utilizing the command: humann2_renorm_table. Following this, gene families were then regrouped from Uniref90 to KEGG (Kyoto Encyclopedia of Genes and Genomes) orthology (KO) using the command: humann2_regroup_table. Unbiased metagenomics assessment was conducted using the MicrobiomeAnalyst software on the converted HUMAnN 2.0 output data. The EdgeR feature was utilized for taxonomic and functional assessment, which normalizes read counts followed by low abundance removal and False Discovery Rate (FDR) correction (29). From the overall KO metagenomics set, a comprehensive search was performed for any genes related to the keywords indole or tryptophan. Output from HUMAnN 2.0 pathway analysis was also characterized using the EdgeR feature of MicrobiomeAnalyst in a similar manner for the assessment of pathways related to tryptophan metabolism.

### Data Analysis

Subject demographics and medications were compared between all four groups using ANOVA or Fischer’s exact test. All tests of significance with p-value <0.05 was considered statistically significant. Metabolomics assessment was performed using MetaboAnalyst software (30). PERMANOVA was performed in R. Taxonomic and functional profiling was performed using MicrobiomeAnalyst software. For taxonomy alpha diversity, students t-test was utilized using the methods of Observed, Chao, Shannon, and Simpson. Beta diversity was assessed utilizing Bray-Curtis dissimilarity and visualized with a Principal Coordinate Analysis (PCoA) plot. PERMANOVA was performed using MicrobiomeAnalyst software of the beta diversity clustering. Statistical analysis of previously obtained Assessment of differential abundance of taxa was performed using EdgeR for sparse data correction, and utilizing a p-value cutoff of 0.05 and FDR of 0.1 given low abundance OTUs, where indicated in the results section. Taxonomic data was then log corrected with correction factor of log(x+1) to account for zero values as has previously been described (31). Metagenomics analysis was performed using MicrobiomeAnalyst, and EdgeR was utilized for sparse data correction with an adjusted p-value cutoff of 0.05 and FDR of 0.05. Statistical analyses and graphics were conducted with GraphPad 8.2 (GraphPad Software).

## Results

### Subjects

In total 84 subjects were included in this study: 24 HC, 27 CD, 21 axSpA, and 12 CD-axSpA. Patient demographics are described in **Supplemental Table 1**. Overall groups were not significantly different with regards to age, sex, ethnicity, smoking, and family history of autoimmunity. As expected, a significantly higher prevalence of HLA-B27 was detected in axSpA groups. Given that subjects with overlapping CD-axSpA would be treated for one or the other condition at the time of the second disease diagnosis, obtaining newly diagnosed, untreated subjects was not feasible. Therefore, TNF-inhibitor (TNFi) usage was matched across the three disease groups. All subjects underwent fecal sampling and either colonoscopy or sigmoidoscopy with biopsies as described in the Methods. After analysis for macroscopic and histologic intestinal inflammation in the subjects, gross inflammation in the terminal ileum and histologic evidence of CD was noted in one subject recruited to the axSpA group who, therefore, was recategorized into the CD-axSpA group.

### Bacteria-Produced Indoles are Increased in Axial Spondyloarthritis Colon Tissue

**Figure 1:**
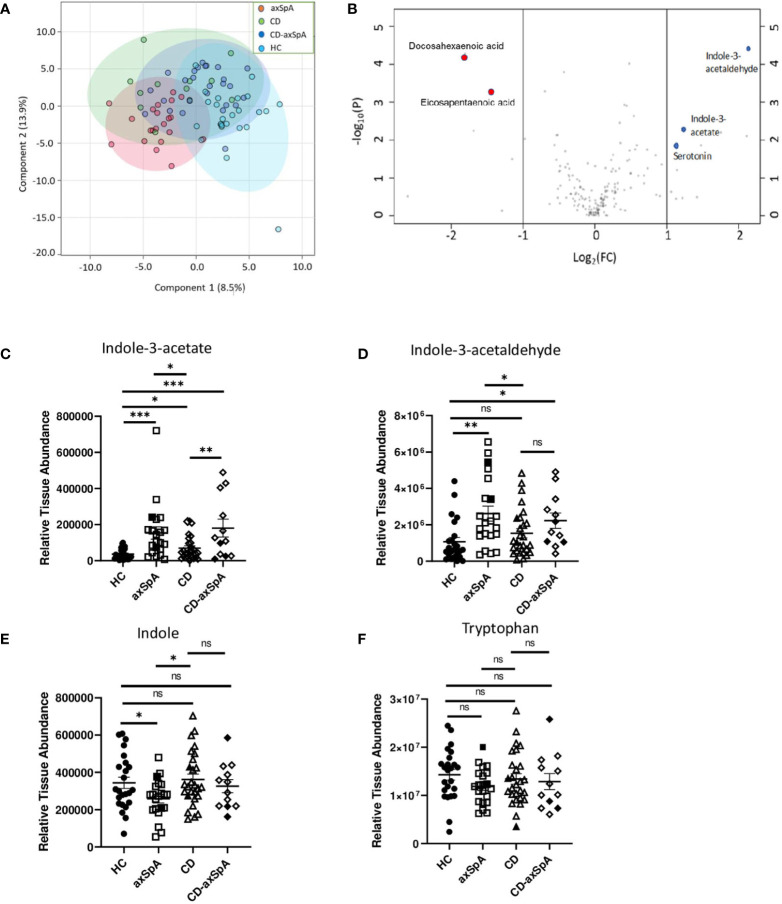
Bacteria-produced indoles are significantly increased in axSpA. Broad screening of metabolites was performed by LC-MS in colon biopsies from HC, axSpA, CD, and CD-axSpA. **(A)** PCoA of the metabolite data in all four groups is shown. By PERMANOVA, p<0.001. **(B)** Volcano plot comparing the fold change differences in metabolites (x-axis) between HC and axSpA versus p-value (y-axis). Positive fold change values indicate higher in axSpA while negative values are those higher in HC. Blue dots represent identified tryptophan derivatives of significance, and red dots represent omega-3 metabolites. Relative tissue concentrations for **(C)** IAA, **(D)** I3Ald, **(E)** indole, and **(F)** tryptophan in each subject is indicated by symbols with bars as the mean ± SEM, measures relative to one another after using the same starting tissue weight. Open symbols represent those subjects taking TNFi. *p < 0.05, **p < 0.005, ***p < 0.0005, and ns (not significant) were determined by ANOVA with Kruskal-Wallis post-hoc test.

We first sought to assess the relevant bacterial metabolites that are taken up by the host in axSpA compared to HC and CD. Therefore, colon biopsies underwent broad assessment of central energy and redox metabolites, yielding 184 named metabolites by LC-MS (**Supplemental Figure 1** and **Supplemental Table 2**). Principal components analysis (PCoA) demonstrated significant separation between axSpA and HC groups but overlap within CD and CD-axSpA (p<0.001 by PERMANOVA; **Figure 1A**). Using a VIP plot to identify the top factors driving the separation in the PCoA, several metabolites within the omega-3 fatty acid and amino acid pathways, including tryptophan derivatives, were identified (**Supplemental Figure 2**). Additionally, using Venn diagrams to demonstrate the similarities and differences from pairwise comparisons of the most significantly changed metabolites, we discovered that indole-containing compounds from tryptophan metabolism associated with the presence of axSpA when compared to HC; when compared to CD, the presence of axSpA seemed to associate with omega 3 fatty acids (**Supplemental Figure 3**). Using a volcano plot to demonstrate which metabolites were most significantly different between axSpA and HC, three indole-containing byproducts of tryptophan metabolism were identified as significantly increased in axSpA relative to HC while omega 3 compounds were significantly decreased (**Figure 1B**).

Of the two major pathways affected in our analysis, two of the three byproducts of tryptophan metabolism, indole-3-acetate (IAA) and indole-3-acetaldehyde (I3Ald), are hypothesized to be microbially generated (32–34), making them intriguing products of the gut microbiome. Relative colon tissue levels of these two metabolites demonstrate significant increases in IAA in both axSpA and CD-axSpA when compared to HC and CD (**Figure 1C**). I3Ald was also significantly elevated in axSpA compared to HC and CD; this metabolite was significant also in CD-axSpA versus HC and elevated with a trend towards significance in comparison to CD (**Figure 1D**). For comparison, the parent compounds tryptophan and indole are included, and only demonstrate a significant difference with a decrease in concentration for indole in axSpA compared to HC and CD (**Figures 1E, F**). In sum, these data allude to altered tryptophan metabolism by the microbiome in the setting of axSpA that is not driven by the presence of bowel inflammation.

### Taxonomic Classification in Axial Spondyloarthritis Exhibits Minimal Evidence of Gut Dysbiosis

**Figure 2:**
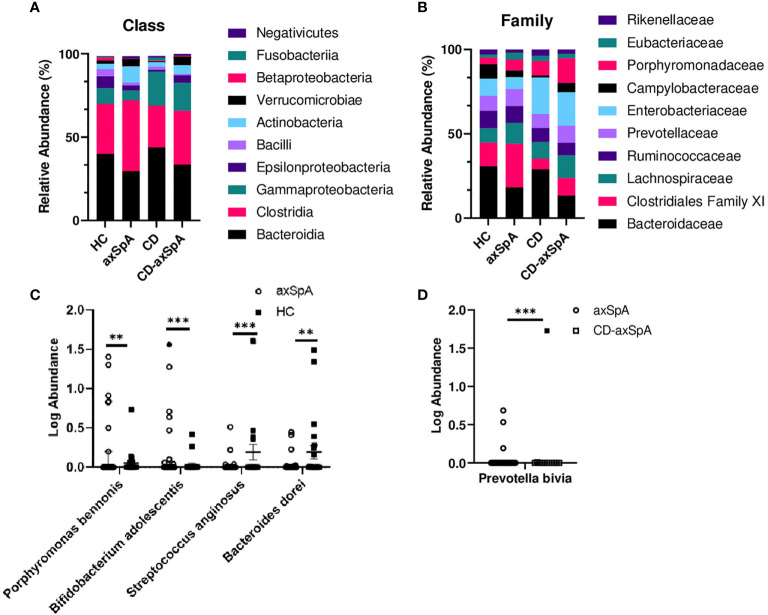
Minimal taxonomic differences are observed in axSpA versus HC. Shotgun metagenomics were performed on rectal swabs from study subjects. The relative abundance of bacterial taxa at the **(A)** class and **(B)** family levels are shown as stacked bars of the mean taxa abundance within the subject group. No significant difference (p>0.05) was detected as determined by ANOVA with Kruskal-Wallis post-hoc test. **(C)** Log transformed relative abundances of individual species in the axSpA vs. HC groups and **(D)** axSpA vs. CD-axSpA groups are shown as symbols for each individual, with open symbols representing those subjects taking TNFi. Bars represent the median relative abundance ± interquartile range. **p < 0.005 and ***p < 0.0005 as determined by Kruskal-Wallis post-hoc test.

We next sought to identify bacterial associations with the highly defined subject groups in our study using shotgun metagenomics, focusing on the differences between HC and axSpA given their striking metabolic findings. An average of 7,874,610 ± 1,765,741 paired-end reads were obtained from the axSpA subjects, 23,458,021 ± 9,039,626 paired end reads from the CD subjects, 23,982,607 ± 7,473,020 from the CD-axSpA, and 8,487,020 ± 874,485 from the HC subjects. After data processing and quality control with Kneaddata 0.7.5, a total of 1,448,835 ± 2,043,378 paired-end reads were obtained from the axSpA subjects, 1,822,197 ± 2,069,588 paired end reads from the CD subjects, 2,291,875 ± 2,184,367 from the CD-axSpA, and 1,121,841 ± 1,276,699 from the HC subjects (**Supplemental Table 3**). Taxonomic profiling was performed using the MicrobiomeAnalyst software. While there were no statistical differences at class and family taxonomic levels across groups (**Figures 2A, B**, respectively), in pairwise comparisons, two species were found to be significantly higher in axSpA: *Bifidobacterium adolescentis* (p<7.22x10^-4^, FDR 0.055) and *Porphyromonas bennonis* (p<0.001, FDR 0.055), and two species were found to be higher in HC: *Streptococcus anginosus* (p<1.76x10^-4^, FDR 0.028) and *Bacteroides dorei* (p<0.001, FDR 0.055) (**Figure 2C**). Alpha diversity analyses of richness and evenness as assessed by Observed, Chao1, Shannon, and Simpson indices were not different between the four subject groups (**Supplemental Figures 4A–D**). Beta diversity was assessed by PCoA on the basis of axSpA vs HC using Bray-Curtis dissimilarity and PERMANOVA, and found to have no separation (**Supplemental Figure 4E**). Given that previous studies included subjects with CD-axSpA in their microbiome analyses of axSpA (6, 7), we compared axSpA to CD-axSpA (**Figure 2D**) and found only one species to be different between the two groups, which was higher in the CD-axSpA group, *Prevotella bivia* (mean axSpA 0.059/median 0, mean CD-axSpA 0.145/median 0, p<3.0x10^-4^, FDR 0.051). Further comparisons were performed for all other groups to understand underlying taxonomic differences between the groups based upon the presence of bowel inflammation using an FDR cut-off of 0.1 (**Supplemental Figure 5** and **Supplemental Table 4**). There were no species level differences between CD-axSpA and HC (data not shown). Because prior microbiome studies compared axSpA and CD-axSpA combined versus HC as stated above, we performed this analysis with our data set and found significantly increased abundances of *Porphyromonas bennonis* (p<0.002, FDR 0.072) as well as decreased abundances of *Prevotella buccalis* (p<0.002, FDR 0.072), *Streptococcus anginosus* (p<5.12x10^-6^, FDR 9.59x10^-4^), *Bacteroides dorei* (p<9.11x10^-5^, FDR 0.008), *Bacteroides thetaiotamicron* (p< 0.001, FDR 0.066), and *Sutterella wadsworthensis* (p<0.002, FDR 0.072) in the combined axSpA groups (**Supplemental Figure 5A**). *Fingoldia magna* (p<3.30x10^-4^, FDR 0.021*)* and *Akkermansia muciniphilia* (p<3.48x10^-4^, FDR 0.021) were significantly expanded in axSpA compared to CD while *Bacteroides dorei* (p<1.88x10^-4^, FDR 0.021) was more abundant in CD versus axSpA (**Supplemental Figure 5B**). *Akkermansia muciniphilia* (p<1.15x10^-4^, FDR 0.019) as well as *Porphyromonas somerae* (p<8.97x10^-4^, FDR 0.077) were more abundant in CD-axSpA compared to CD (**Supplemental Figure 5C**). When CD was compared to HC, *Campylobacter hominis* (p<8.88x10^-5^, FDR 0.014) and *Streptococcus anginosus* (p<7.49x10^-4^, FDR 0.058) were significantly more abundant in subjects with HC (**Supplemental Figure 5D**).

Lastly, previously published disease-relevant species of *Ruminococcus gnavus*, *Dialister invisus*, *Akkermansia muciniphila*, and *Eschericia coli* (4–7) were compared against all four groups (**Supplemental Figures 6A–D**, respectively). Of these comparisons, only with *Dialister invisus* were we able to replicate the observations of Tito et al. (7), which was significantly increased in abundance in subjects with axSpA and CD-axSpA, but not CD, compared to HC (**Supplemental Figure 6B**), supporting the specificity of this taxa for axSpA.

### Metagenomic Profiling Identifies Altered Tryptophan Metabolism in Axial Spondyloarthritis

Finally, in order to link the bacterial changes in axSpA to our observations of increased IAA and I3Ald from metabolomics screening, we analyzed gene abundance and pathway data from the bacterial sequencing. Taxonomic and functional data from HUMAnN 2.0 was merged among all samples appropriately for pairwise comparisons, then run in MicrobiomeAnalyst using EdgeR software. MicrobiomeAnalyst software takes read per kilobase output from HUMAnN 2.0, converts to copies per million, and streamlines into EdgeR input format. EdgeR was initially developed for RNA-seq analysis, but is effective for the analysis of metagenomics data due to challenges with sparse data analysis utilizing traditional statistics methods (29, 35). The software normalizes total read counts, filters low abundance features, then calculates the log_2_ fold change between groups using the Benjamini-Hochberg false discovery rate (FDR) test with a threshold of 0.05. Diversity analysis of the complete data set was performed across KEGG metabolic pathways looking at a global representation of all associated genes in their appropriate metabolic pathways. No statistical difference was noted in the global pathway analysis representing KEGG metabolic pathways between axSpA and HC (**Supplemental Figure 7A**). PCoA was also visualized on the basis of the metagenomics data displaying no difference between groups (**Supplemental Figure 7B**), as well as a dendrogram of all samples present showing no notable separation (**Supplemental Figure 7C**). Thus, in a global survey of the metagenomics data, we did not identify functional genetic pathways that differed between axSpA and HC.

HUMAnN 2.0 pathway analysis was then performed to identify significantly altered gene function pathways between HC and axSpA, providing additional insight into the overall functional metagenomic status. These data were analyzed as above with the gene level data using the EdgeR feature of MicrobiomeAnalyst, and 61 pathways meeting the criteria of p<0.05 and FDR<0.05 are displayed in **Supplemental Table 5** after removal of individual species level pathways; the superpathway of L-tryptophan biosynthesis was identified as significantly increased in HC compared to axSpA. No omega 3 fatty acid pathway was identified. Because our metabolic data heavily implicated changes in tryptophan metabolism by bacteria, a search was then performed for all pathways involved in tryptophan metabolism, without omission of individual species level pathways. Three pathways related to tryptophan biosynthesis were identified, all of which were higher in HC (**Table 1**).

**Table 1:** Composite tryptophan pathway analysis of metagenomic data.

Pathway	log_2_FC (HC:axSpA)	P-value	FDR
PWY-6629: superpathway of L-tryptophan biosynthesis	2.1065	0.002069	0.012391
TRPSYN-PWY: L-tryptophan biosynthesis *Escherichia coli*	1.6584	0.002245	0.01312
PWY-6629: superpathway of L-tryptophan biosynthesis *E. coli*	1.5871	0.004639	0.021526

After running the complete metagenomics dataset at a pathway level, we then identified gene level data that significantly differed in relative abundance between axSpA and HC (using p<0.05 and FDR<0.05). Without a clear pathway linking these genes from our pathway analysis, we focused on the tryptophan pathway given our metabolic data strongly associating its indole metabolites with axSpA. A search was performed for individual genes associated with the keywords indole or tryptophan. A total of 35 individual genes were found to be statistically significant based upon the cutoff of p<0.05 and FDR<0.05 and are displayed in **Table 2**. Values in which the log_2_ fold change are positive are considered higher in HC while negative values are higher in axSpA (displayed in gray). Within the tryptophan metabolic pathways, 18/27 genes relevant to tryptophan synthesis were significantly more abundant in HC while 2/2 genes for tryptophan metabolism were significantly more abundant in axSpA (**Table 2**), suggesting that in axSpA, the bacterial community shifts from tryptophan synthesis to metabolism.

**Table 2:** Relative tryptophan pathway gene abundances in HC vs. axSpA bacterial metagenomics.

Gene	log_2_FC (HC:axSpA)	P-value	FDR	Trp Function
K00179: indolepyruvate ferredoxin oxidoreductase, alpha subunit *Bacteroides eggerthii*	−2.7458	1.31E-05	0.001179	Unclear
K00179: indolepyruvate ferredoxin oxidoreductase, alpha subunit *Alistipes finegoldii*	1.5259	0.001195	0.012517	Unclear
K00180: indolepyruvate ferredoxin oxidoreductase, beta subunit *A. finegoldii*	1.6765	0.000462	0.007469	Unclear
K00180: indolepyruvate ferredoxin oxidoreductase, beta subunit *Bacteroides ovatus*	−1.36	0.008443	0.042738	Unclear
K00180: indolepyruvate ferredoxin oxidoreductase, beta subunit *B. fragilis*	1.3126	0.004987	0.02983	Unclear
K01609: indole-3-glycerol phosphate synthase *Roseburia intestinalis*	−2.478	2.33E-05	0.001495	Synthesis
K01609: indole-3-glycerol phosphate synthase unclassified	−2.0789	0.00066	0.008987	Synthesis
K13498: indole-3-glycerol phosphate synthase/phosphoribosylanthranilate isomerase *Escherichia coli*	1.6051	0.001603	0.014665	Synthesis
K13498: indole-3-glycerol phosphate synthase/phosphoribosylanthranilate isomerase	1.5257	0.002535	0.019341	Synthesis
K01609: indole-3-glycerol phosphate synthase *Akkermansia muciniphila*	2.1624	0.002691	0.020043	Synthesis
K01609: indole-3-glycerol phosphate synthase *B. fragilis*	1.4215	0.003307	0.022733	Synthesis
K04103: indolepyruvate decarboxylase	−1.2603	0.004983	0.029816	Metabolism
K04103: indolepyruvate decarboxylase *Corynebacterium aurimucosum*	−1.1923	0.008084	0.041395	Metabolism
K01667: tryptophanase *B. thetaiotaomicron*	1.9251	3.58E-05	0.001874	Synthesis
K01667: tryptophanase *A. finegoldii*	1.4811	0.001467	0.013997	Synthesis
K07185: tryptophan-rich sensory protein *A. finegoldii*	1.8725	0.000181	0.004631	Signaling
K01867: tryptophanyl-tRNA synthetase *E. coli*	2.2716	0.000231	0.005242	Synthesis
K01867: tryptophanyl-tRNA synthetase *Ruminococcus bromii*	−2.1286	0.000352	0.006525	Synthesis
K01867: tryptophanyl-tRNA synthetase *R. intestinalis*	−1.5828	0.002507	0.019205	Synthesis
K01867: tryptophanyl-tRNA synthetase *B. thetaiotaomicron*	1.5689	0.003252	0.022486	Synthesis
K01867: tryptophanyl-tRNA synthetase *Alistipes shahii*	−1.2245	0.007021	0.037604	Synthesis
K01867: tryptophanyl-tRNA synthetase *A. muciniphila*	1.6871	0.009087	0.045048	Synthesis
K01695: tryptophan synthase alpha chain *E. coli*	1.6638	0.00159	0.014609	Synthesis
K01695: tryptophan synthase alpha chain *R. bromii*	−1.6939	0.002344	0.018449	Synthesis
K01695: tryptophan synthase alpha chain *A. muciniphila*	1.7194	0.006817	0.036817	Synthesis
K01696: tryptophan synthase beta chain *R. intestinalis*	−1.982	0.000272	0.005681	Synthesis
K01696: tryptophan synthase beta chain *A. muciniphila*	2.1602	0.000671	0.009074	Synthesis
K01696: tryptophan synthase beta chain *Ruminococcus lactaris*	1.5981	0.000789	0.009917	Synthesis
K06001: tryptophan synthase beta chain *A. muciniphila*	1.8548	0.002536	0.019342	Synthesis
K01696: tryptophan synthase beta chain *B. fragilis*	1.3137	0.004084	0.026096	Synthesis
K06001: tryptophan synthase beta chain *R. intestinalis*	−1.4264	0.004439	0.02757	Synthesis
K01696: tryptophan synthase beta chain *R. bromii*	−1.496	0.008027	0.041207	Synthesis
K01696: tryptophan synthase beta chain *Streptococcus anginosus*	1.1735	0.009845	0.047575	Synthesis
K02846: N-methyl-L-tryptophan oxidase	1.7694	0.001406	0.013714	Synthesis
K02846: N-methyl-L-tryptophan oxidase *E. coli*	1.7694	0.001406	0.013714	Synthesis

**Figure 3:**
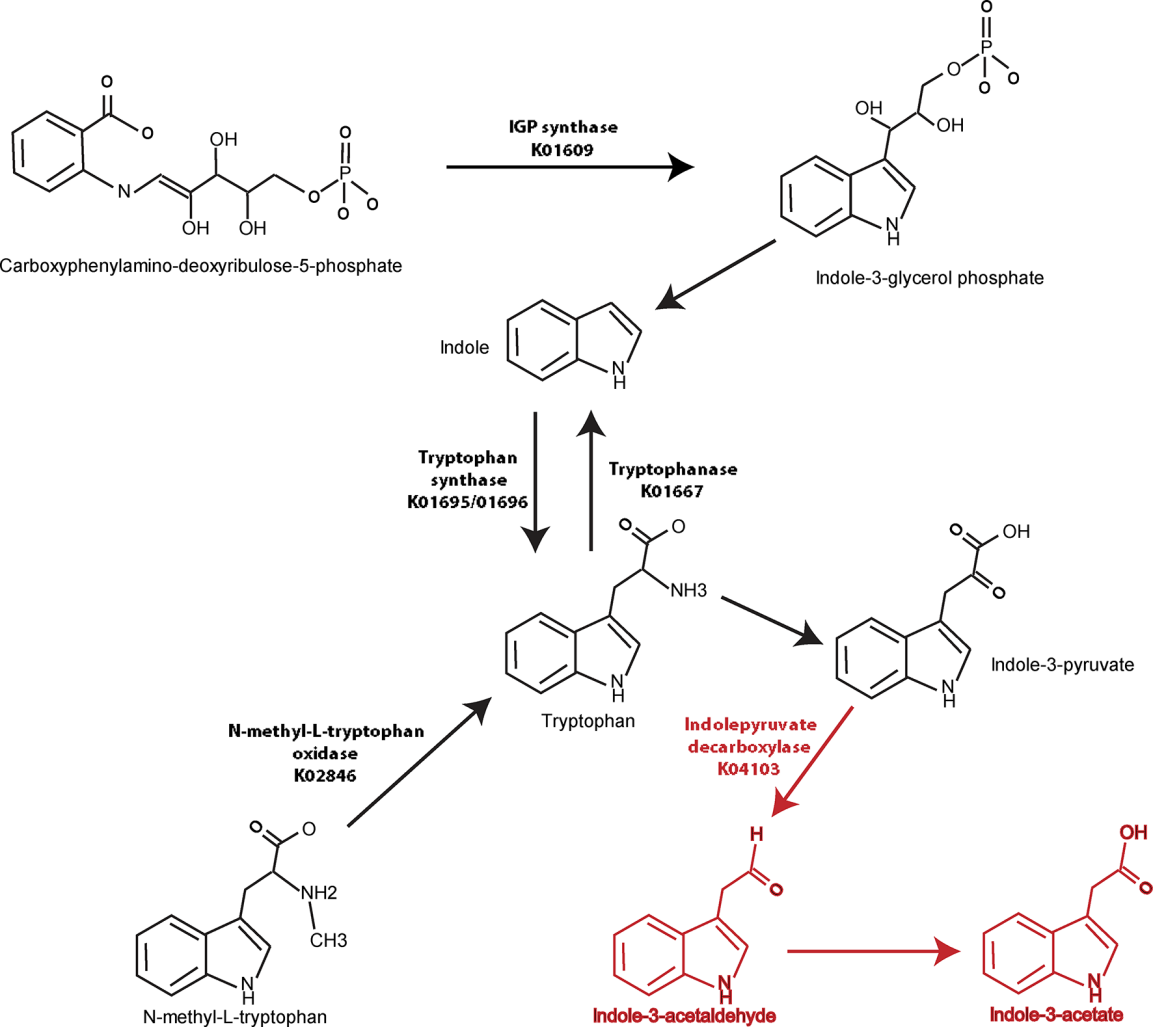
Graphical demonstration of identified tryptophan pathway gene differences relative to generated metabolites in axSpA and HC. Genes encoding enzymes in the tryptophan synthesis/metabolism pathway were identified using metagenomics (**Table 2**) and are represented visually here. Numerous pathways towards tryptophan synthesis were identified as significantly higher in HC compared to axSpA and shown with black arrows and listed KO names. Indolepyruvate decarboxylase, which is responsible for tryptophan metabolism towards IAA and I3Ald, was found to be significantly increased in genetic abundance in axSpA compared to HC. This gene and the resulting metabolites that are increased in axSpA are identified in red.

Similar analyses were performed in a pairwise manner among remaining groups and demonstrated in **Supplemental Tables 6–10**. When the axSpA and CD-axSpA groups were combined, 13/27 genes relevant to tryptophan synthesis were more abundant in the HC group (**Supplemental Table 6**), suggesting that the microbiome in the presence of bowel inflammation increases tryptophan synthesis. Indeed, tryptophan synthesis genes were overwhelmingly increased in abundance in CD-axSpA compared to axSpA (**Supplemental Table 7**) and in CD compared to HC (**Supplemental Table 8**). However, the gene encoding indolepyruvate decarboxylase, the enzyme that metabolizes tryptophan products to IAA, remained more abundant in the combined axSpA and CD-axSpA comparison to HC (**Supplemental Table 6**) as well as in CD-axSpA compared to HC (**Supplemental Table 9**) and CD-axSpA compared to CD (**Supplemental Table 10**), suggesting that this pathway is specific to axSpA regardless of bowel inflammation. A composite model describing gene abundances in the tryptophan pathway relative to disease status is illustrated in **Figure 3**.

## Discussion

The lack of consensus taxa across multiple studies associating intestinal dysbiosis with axSpA raises the question of relevance to disease pathophysiology. Rather, based on studies in HLA-B27 transgenic rats and emerging data in PsA (3, 17, 18), we hypothesized that the microbial population as a whole may act through bacterially produced metabolites. In this study, we utilized unbiased approaches to characterize and connect gut metabolomics and bacterial metagenomics in axSpA. Screening across central metabolism and redox metabolites by LC-MS in which we were able to detect 184 metabolites, we identified significantly increased indole-containing metabolites of the tryptophan pathway associated with the presence of axSpA with or without confounding bowel inflammation. We also noted several other metabolic pathways that were affected, such as a significant reduction in omega-3 fatty acids that associated with axSpA. Through profiling of the bacterial metagenome, we identified numerous pathways that were altered in subjects with axSpA compared to HC. However, only the tryptophan pathway was consistent in both the metabolomic and metagenomic data sets. Given this overlapping finding, we confirmed tryptophan pathway alterations in our subjects. Altogether, our findings demonstrate that metabolism of tryptophan by the microbiome into indole derivatives to be of significance in axSpA.

Complimentary to our findings, others have shown tryptophan to be decreased in the plasma in axSpA (36), although other studies utilizing independent cohorts and different methods of metabolomic detection (NMR, LC-MS, GC-MS, etc.) have not replicated this finding (37–39). In the feces, one study found decreased cholesterols and steroids in subjects with AS compared to controls (40) suggesting these pathways, including our findings of omega-3 fatty acid alterations, should be further pursued in future studies. However, in juvenile enthesitis-related arthritis, two independently studied cohorts demonstrated reduced tryptophan metabolism in the feces in spite of the lack of taxonomic differences in the microbiomes of these cohorts compared to controls (41). A limitation to these fecal studies, though, is that fecal metabolites may not reflect what the host absorbs, which is the reason why we performed a metabolic analysis on intestinal tissue. To our knowledge, such an analysis has not been performed previously.

Although the pathophysiologic role of tryptophan metabolism and indole within the intestine in axSpA will need to be demonstrated, an accumulating literature supports its role in local epithelial barrier and immune cell function [reviewed in (32–34)]. Tryptophan is solely derived from dietary intake and absorbed by the host for use in protein synthesis and other metabolic pathways, particularly kynurenine and serotonin derivatives. While bacteria can metabolize tryptophan to kynurenine, they also metabolize dietary tryptophan into indole and using the enzyme tryptophanase as well as other indole derivatives; the host does not generate indole as tryptophanase is exclusive to bacteria (33, 34). Indole-containing derivatives, which can be produced by a variety of microbes, plants, and even recently observed by a human cancer cell line (42), are absorbed across the intestinal epithelium of the host and signal through either the aryl hydrocarbon receptor (AhR) or the pregnane X receptor (PXR) to modulate host responses including barrier and immune functions. Indole and its derivatives have varied effects on the host from promotion of inflammatory responses to regulation and resolution of inflammation depending upon the specific metabolite, receptor, cell, and experimental model (32). For example, in lupus-prone mice, dysbiosis is linked to altered tryptophan catabolism, and feeding a high tryptophan diet correlates with worse disease and greater autoantibody generation (43). Yet, in experimental autoimmune encephalitis, tryptophan metabolism, and specific indole-containing derivatives were linked to reduced CNS inflammation (44). Specifically within the intestine, indole-containing derivatives such as indole-3-propionic acid (IPA) signals directly through epithelial cells to maintain and repair the barrier (45) while IAA and I3Ald, through AhR (14, 46, 47) signaling on innate lymphoid cells, results in increased IL-22 expression in the gut mucosa (46). Thus, there are wide-ranging effects of tryptophan metabolism and its indole-containing derivatives. Although not the main objective of the study, taxonomic profiling in this study failed to reveal differences in alpha or beta diversity by multiple measures (**Supplemental Figures 4A–E**) or in higher order OTUs between the axSpA and HC groups (**Figures 2A, B**). Such a result is likely due to the use of TNFi in our subjects, as TNFi has previously been shown to “normalize” the microbiome in axSpA (47–49). To further assess the role of specific bacterial differences across axSpA, a comparison was made across the most previously published species in axSpA (**Supplemental Figure 6**) **(** 4, 6, 7). Within our cohort, the most significantly associated species was *D. invisus*, which had a significant expansion in both the axSpA and CD-axSpA groups. *R. gnavus* had a decreased abundance in axSpA, and *A. muciniphilia* and *E. coli* were not statistically significant across groups but trended towards previously published results. Put together, these data suggest similarities within our cohort to those previously published, in spite of the limitation of TNFi use by our cohort.

Our evaluation of bacterial metagenomics between axSpA and HC identified a number of tryptophan metabolism pathways that are altered. In general, our data suggest the microbial community in HCs increased tryptophan synthesis (or decreased synthesis in the axSpA group), while the microbial community in axSpA increased tryptophan metabolism towards indoles (**Figure 3**). Analysis through HUMAnN 2.0, which evaluates global pathways rather than individual genes, also demonstrates increased tryptophan synthesis in HC relative to axSpA (**Table 1**). These metagenomics analyses are consistent with the identification of increased indole derivates through metabolomic screening in the gut tissue of patients with axSpA (**Figure 1**) (33). The combined observations could represent a functional difference of the axSpA microbiome. This is especially noteworthy given the lack of dysbiosis observed within the microbiome, implying that the shift in tryptophan metabolism is not specific to the particular dysbiosis in axSpA and perhaps more of a generalized community function as has previously been suggested (10).

While we focus our metagenomics conclusions on the tryptophan pathway, due to the consistency with our metabolomics analyses, we do observe similar metagenomics pathways in comparison with previously published results. For example, in agreement with other’s findings of the TCA cycle and biotin synthesis being enriched in AS, and butanoate pathways and pyridoxal 5’-phosphate salvage pathways being enriched in controls (47, 50, 51). These studies focused on untreated AS, but when comparing untreated to TNFi treated AS cases, not only did the microbiome become less significantly different from controls, so did metagenomic findings (47). Intriguingly, the authors of this particular study found that aromatic amino acids, which includes tryptophan, synthesis was significantly reduced in untreated AS cases compared to both treated AS cases and controls (47). This may indicate that our findings, where arguably our AS subjects were not fully controlled based on BASDAI scores, have a smaller effect due to TNFi treatment that would be otherwise more pronounced in the absence of this confounder.

One key identified gene was indolepyruvate decarboxylase, which encodes for the enzyme that converts indole-3-pyruvate to IAA and I3Ald (52), and was increased in axSpA. Other genes encode for different aspects of tryptophan synthesis such as tryptophan synthase (alpha and beta chain) (53) (elevated in HC), indole-3-glycerol phosphate synthase (54) (elevated in HC), and *N*-methyl-L-tryptophan oxidase (55) (elevated in HC). Tryptophanase is involved in converting tryptophan to indole, which is considered a separate pathway directed away from IAA and I3Ald (56) (elevated in HC). Other findings are of unclear importance due to lack of current published knowledge such as indolepyruvate ferredoxin oxidoreductase, which converts indole-3-pyruvate to *S*-2-(indol-3-yl)acetyl-CoA (elevated in HC). The product of this reaction has unclear biologic function, but presumably can function in immune signaling by acting through the AhR. Tryptophanyl tRNA synthetase engages in protein synthesis through the propagation of protein elongation by adding tryptophan (57), and was found to be elevated in HC, which is consistent with our findings of tryptophan synthesis in HC.

Combining axSpA and CD-axSpA together as one group compared to HC weakened the trend towards increased tryptophan synthesis in the HC group but preserved tryptophan metabolism by indolepyruvate decarboxylase (**Supplemental Table 6**), suggesting that bowel inflammation could promote tryptophan synthesis but metabolism to indoles was specific to axSpA. In support of this conclusion, metagenomic differences in tryptophan metabolism between axSpA and CD-axSpA skewed significantly increased towards tryptophan synthesis in the setting of bowel inflammation, which was similar to CD vs HC (**Supplemental Tables 7** and **8**, respectively). Furthermore, CD and CD-axSpA were similar with regards to tryptophan synthesis (**Supplemental Tables 9** and **10**, respectively). When axSpA, CD-axSpA, or the two groups were combined, indolepyruvate decarboxylase was significantly represented compared to HC or CD, supporting the conclusion that this pathway of tryptophan metabolism is specific to axSpA.

## Limitations

This study has a number of limitations to be addressed. First, the majority of subjects recruited in each of the three cohort groups had been on TNFi therapy. This limitation has been discussed above, and it is intriguing that our metagenomics data still correlates with alterations in the tryptophan metabolism pathway despite a lack of dysbiosis. Repeating this study on a cohort of newly diagnosed, untreated patients would be of interest to validate findings; however, a comparison CD-axSpA group would not be possible due to one or the other disease being diagnosed and treated prior to the development of the other disease.

Sample size in our cohort is somewhat small in general, making definitive statistical conclusions about the microbiome less universal, but our findings still hold true across the different disease states. Additionally, while our cohort purposefully included subjects that were HLA-B27 negative axSpA and females with axSpA, the numbers were underpowered to analyze separately. Other confounding variables such as disease activity, other medication and NSAID use (that are widespread in the general population), and diet, which is often not accurately assessed even in the setting of validated measures (58–60). Further studies that could separate out these groups would be of interest in understanding sex, genetic, medication and dietary differences, but including them in general gives a better picture of the AS disease state as a whole.

In addition, samples were collected as rectal swabs rather than fecal samples. This is still representative of the overall microbiome, but leads to issues such as decreased data recovery using shotgun metagenomics after removal of low abundance data (61). This leads to a perceived lower amount of species diversity as detailed above. Lastly, all subjects were recruited from a single center, so there is unclear geographic bias among the cohort population.

## Conclusions

Using two separate ‘omics approaches of metagenomics and metabolomics, we identified significant alterations in tryptophan metabolism with increased synthesis in HC and those with bowel inflammation and increased metabolism to indoles in the setting of axSpA. Within our study, we uniquely compare axSpA without bowel inflammation, CD, and overlapping CD-axSpA, allowing us to dissect the effects of bowel inflammation and axSpA in our observations. Although our cohort was likely influenced by the use of TNFi, subjects in the axSpA groups had elevated BASDAI scores, implying active disease. Despite the minimal evidence of dysbiosis across our groups, we still noted significant alterations in tryptophan metabolism by both metabolomic assessment and metagenomics analysis. Such findings potentially represent a dysbiotic community effect that has significant implications for host immune function and supports the use of multi ‘omics approaches to identify possible pathways linking the microbiome to pathophysiologic relevance in disease.

## Data Availability Statement

The raw data supporting the conclusions of this article will be made available by the authors, without undue reservation. Sequencing data is publicly assessable in the National Library of Medicine’s Sequence Read Archive SUB8755981.

## Ethics Statement

The studies involving human participants were reviewed and approved by the Colorado Multiple Institutional Review Board. The patients/participants provided their written informed consent to participate in this study.

## Author Contributions

EHR and KAK conceptualized and designed the study. EHR, MEG, BPF, FIS, and AEF recruited the subjects. EHR, MEG, BPF, FIS, and AEF acquired the samples. AJB, AB, AS, and JAR processed and extracted the samples. AJB, AS, JAR, and KAK analyzed and interpreted the data. AJB and KAK drafted the manuscript with input from AS and JAR. All authors contributed to the article and approved the submitted version.

## Funding

Support for the individuals and studies reported here include NIH awards T32AR007534 (AJB and EHR), R01AR075033 and U01AI130830 sub-award, and the Spondyloarthritis Association of America (KAK).

## Conflict of Interest

The authors declare that the research was conducted in the absence of any commercial or financial relationships that could be construed as a potential conflict of interest.
