# Supplementary material for: Multi ‘Omics Analysis of Intestinal Tissue in Ankylosing Spondylitis Identifies Alterations in the Tryptophan Metabolism Pathway
Source: Front Immunol. 2021 Mar 3;12:587119. doi: 10.3389/fimmu.2021.587119 (PMC7966505; doi:10.3389/fimmu.2021.587119)
Supplement: Supplementary file 1 [file Image_1.pdf]

Supplemental Table 1. Subject Characteristics.

Condition	Controls (N=24)	CD (N=27)	axSpA (N=21)	CD-axSpA (N=12)	P-values
**Age in years, mean (SD)**	45.16 (11.8)	35.14 (18.1)	44.92 (12.1)	51.38 (11.1)	0.105
**Male Sex**	50.0%	51.7%	56.5%	38.5%	0.7754
**Non-Hispanic White**	83.3%	86.2%	91.3%	84.6%	0.2917
**Smoker**[a]	21.7%	37.9%	52.2%	53.8%	0.1263
**Family History of Autoimmunity**[b]	16.7%	27.6%	39.1%	46.2%	0.203
**Disease Duration years (SD)**	n/a	11.0 (8.3)	9.9 (9.8)	11.9 (8.0)	0.533
**HLA-B27 positivity**	1 (6.25%)	7 (24.1%)	19 (82.6%)	8 (61.5%)	**9.536×10^{-6}**
**TNF inhibitor Usage**	0%	93.1%	91.3%	69.20%	**3.669×10^{-13}**
**SCCAI**[c]	1.12 (1.2)	2.76 (1.9)	3.26 (1.4)	3.62 (1.5)	0.172
**Harvey Bradshaw Index**	1.17 (1.3)	2.9 (2.7)	3 (1.5)	3.62 (1.4)	0.412
**BASDAI**[d]	1.35 (1.4)	2.86 (2.5)	4.76 (2.4)	5.14 (2.2)	0.167

[a]Smoker defined by ever smoker

[b]Family history of autoimmunity defined based upon the question: “Do you have any first degree relatives (mother, father, sister, brother, or children) with a diagnosis of inflammatory bowel disease (Crohn’s disease/ulcerative colitis), ankylosing spondyloarthritis, psoriasis, or any other autoimmune disease?”

[c]SCCAI, Simple Clinical Colitis Activity Index

[d]BASDAI, Bath Ankylosing Spondylitis Disease Activity Index

Supplemental Figure 1

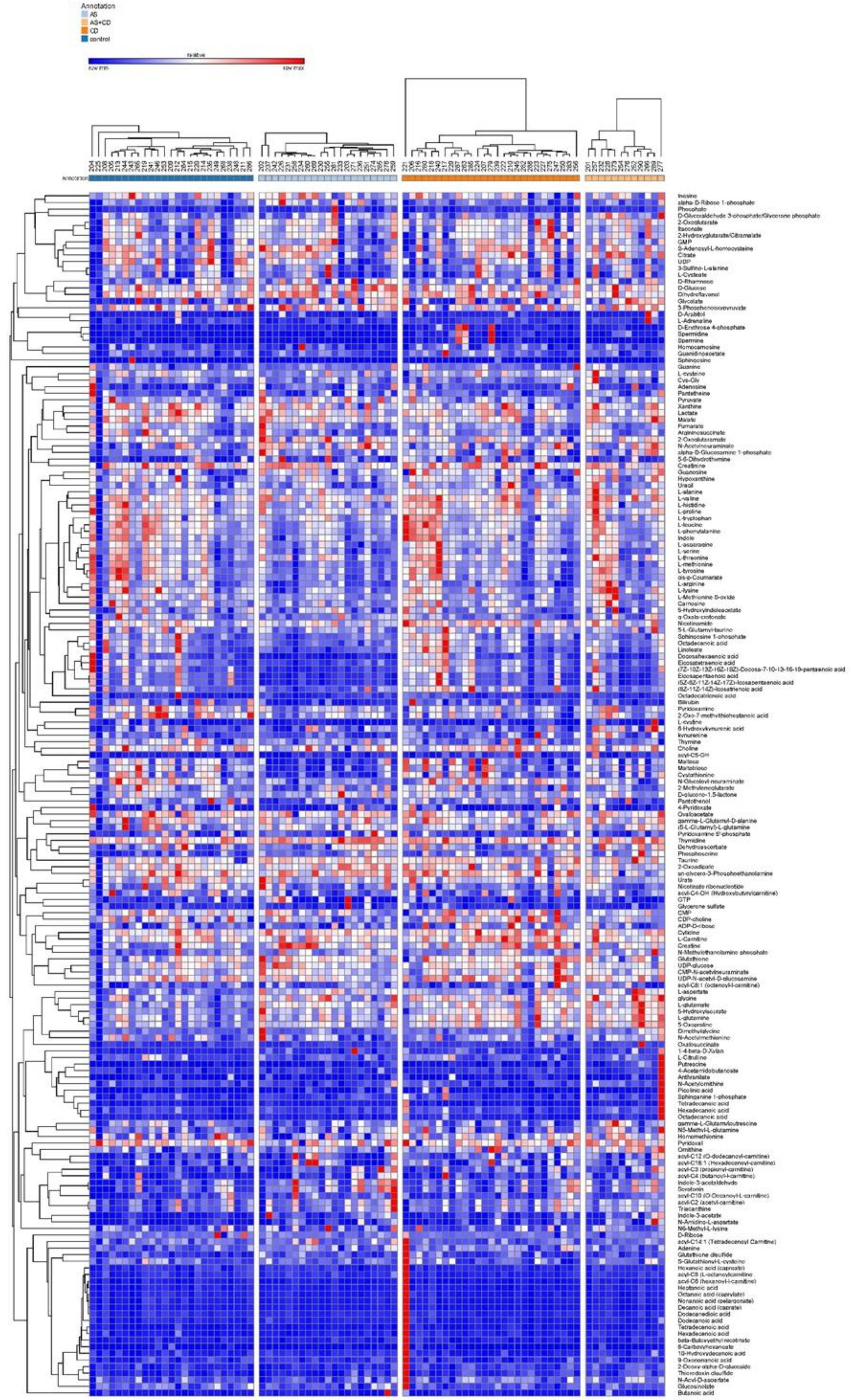

Supplemental Figure 1. Unbiased metabolomics assessment of colon tissue across disease states. Biopsy samples from groups HC, axSpA, CD, and CD-axSpA were evaluated by LC-MS detection of 184 metabolites. Data are the relative tissue abundance (low = blue; high=red) of the specific metabolites listed along the right y-axis for each individual (column) and grouped and analyzed separately by disease state.

Supplemental Table 2. Metabolite concentrations across groups

Metabolites	Mean ± SEM				ANOVA P value	ANOVA FDR P value
	axSpA	CD-axSpA	CD	HC		
L-alanine	54135449.52 ± 3617674.808	65701672.5 ± 7285877.467	57941590.37 ± 3050586.184	52797327.08 ± 3524559.817	0.32234	0.52487
L-arginine	18815971.9 ± 1231168.591	29566663.33 ± 3418915.737	21861389.63 ± 1369695.515	24949774.58 ± 1629005.184	0.003418	**0.024293**
L-asparagine	2065809.61 ± 180206.4851	2779378.083 ± 254437.0159	2658966.815 ± 183706.0266	2609353.5 ± 177270.2395	0.030752	0.14508
L-aspartate	14893154.95 ± 742393.4308	16848215.33 ± 1516119.959	12691766.41 ± 683580.7671	10637166.08 ± 648325.7822	1.24E-05	**0.000503**
L-cysteine	6530693.905 ± 653657.9025	8641522.25 ± 1207336.123	7546336.174 ± 554146.0145	7637065.042 ± 574274.2158	0.58727	0.78875
L-glutamate	61070161.9 ± 2538484.769	68039537.5 ± 5226725.868	57345680 ± 2837574.274	41933635.42 ± 2506579.518	2.02E-07	**2.02E-05**
L-glutamine	23980137.62 ± 1347227.384	31089795 ± 1766583.828	27462647.78 ± 1268556.141	20737149.46 ± 1286160.314	3.84E-05	**0.000884**
glycine	15362726.81 ± 1127986.252	19139199.17 ± 1436776.464	15813738.15 ± 656765.4758	12877265.96 ± 811933.0325	0.001186	**0.010907**
L-histidine	10957457.43 ± 692664.3641	12855827.33 ± 1286913.286	13603105.81 ± 702334.3838	12563951.58 ± 830642.6216	0.16434	0.35161
L-leucine	113931281.9 ± 9174105.698	150098979.2 ± 16326219.59	150265198.1 ± 12685840.08	161406783.8 ± 12402143.32	0.10488	0.2835
L-lysine	21717673.81 ± 1435264.465	33655356.67 ± 3448867.586	27102965.93 ± 1652716.612	29159550.42 ± 2107924.583	0.005489	**0.036069**
L-methionine	10229798.48 ± 721092.8158	14095359.67 ± 1728542.694	13888653.48 ± 1191426.895	14943193.46 ± 1374718.486	0.075706	0.23268
L-phenylalanine	73946426.67 ± 5483582.245	96034555 ± 11163450.34	99261379.26 ± 8473879.432	106857177.1 ± 8287464.711	0.082783	0.24178
L-proline	46210715.24 ± 2784064.002	55438249.17 ± 5034161.638	52091790.37 ± 2558498.152	49404947.08 ± 3595537.715	0.35397	0.56388
L-serine	4083270.81 ± 289890.1317	4713360.667 ± 487380.0651	4914193.296 ± 334196.9179	5139307.792 ± 365580.0873	0.16434	0.35161
L-threonine	6919225.714 ± 461626.0543	8367477.5 ± 834925.968	7741006.778 ± 414178.2442	7464619.375 ± 534452.3409	0.46118	0.67886
L-tryptophan	11852321.38 ± 803819.472	12860333.58 ± 1672065.498	13414952.56 ± 1066261.359	14294483.96 ± 1094631.295	0.74859	0.89282
L-tyrosine	7674476.19 ± 468185.4365	10301491.33 ± 1192846.852	10149760.74 ± 763977.5326	11264985.63 ± 921531.6475	0.030412	0.14508
L-valine	73386330.95 ± 3970913.228	86177941.67 ± 6986509.265	80942577.04 ± 3864937.842	72829223.75 ± 4136808.911	0.2451	0.43364
L-cystine	64116.37962 ± 10548.97452	248182.776 ± 127899.5029	119633.9655 ± 20378.28345	162681.1605 ± 40998.43591	0.49843	0.70008
Adenosine	1101146.148 ± 148996.392	1108335.833 ± 280623.5991	946328.5037 ± 143569.8227	813219.3208 ± 163932.6842	0.23237	0.42414
Adenine	247434.0129 ± 17634.43741	213442.1167 ± 21194.78343	261931.1444 ± 24214.97055	202729.5125 ± 11835.02558	0.17494	0.36167
GTP	1907602.847 ± 583837.2615	810799.4783 ± 258218.8042	1290487.141 ± 276244.9419	1097957.144 ± 191892.2727	0.14499	0.33349
GMP	839498.95 ± 130150.9218	925955.325 ± 126341.9371	1021907.828 ± 110944.2359	816182.8921 ± 120085.7141	0.47562	0.68908

Guanosine	1123396.614 ± 58831.27567	1240591.575 ± 82277.30451	1107388.196 ± 78334.39973	991597.7083 ± 59336.96594	0.15058	0.34206
Guanine	275043.7476 ± 30182.2579499999	293175.8667 ± 46261.42926	312128.4778 ± 42504.44853	302491.6967 ± 33669.69578	0.99532	0.99532
CMP	1338487.81 ± 100216.9895	1293846.567 ± 92698.05338	1481630.115 ± 113197.3898	1435694.263 ± 83273.74002	0.81874	0.91274
Cytidine	2176846.286 ± 78274.44188	2145462.167 ± 147976.1275	2134677.111 ± 111188.3503	1921524.833 ± 116734.1558	0.21172	0.4058
Thymidine	299426.8476 ± 11533.60929	283922.7333 ± 19380.75019	258198.203 ± 12385.79386	251808.1583 ± 9866.041729	0.11062	0.29077
Thymine	1853092.538 ± 144078.4092	1635217.4 ± 264658.3859	1774754.781 ± 197731.2863	2213586.696 ± 199850.3275	0.23802	0.42938
UDP	384360.1214 ± 47936.19234	334144.6417 ± 53046.01818	413828.2541 ± 50188.4406799999	520277.0321 ± 72866.9035	0.94236	0.97517
Uracil	528041.3571 ± 43708.9687	706362.1667 ± 79747.53276	674137.5963 ± 53953.93123	607522.2 ± 41242.72847	0.080932	0.24055
Inosine	9765501.429 ± 661876.1954	9266988.667 ± 911178.1498	9071875.37 ± 614279.0022	9285595.792 ± 597931.3222	0.82841	0.91274
Hypoxanthine	51419374.76 ± 2172363.155	58759364.17 ± 5341189.415	51012158.89 ± 2349315.247	52872359.17 ± 3288068.922	0.63066	0.81174
Xanthine	8487016.714 ± 641587.1151	9221012.333 ± 840301.0911	8080832.37 ± 541365.8484	8799268.542 ± 679007.6573	0.76646	0.89827
5-Hydroxyisourate	2716980.429 ± 219031.3437	3585368.417 ± 300496.2182	2983858.041 ± 189992.3669	2021157.7 ± 144547.5381	8.18E-05	**0.00131**
Urate	9146092.286 ± 808874.3485	8284833.667 ± 750607.2424	8727666.37 ± 541566.4236	9526078.5 ± 740642.9372	0.94207	0.97517
5-6-Dihydrothymine	9366079.014 ± 1965259.898	5658123.417 ± 1336575.774	9893822.37 ± 1513161.406	5575546.038 ± 1075316.147	0.040056	0.1714
Pyridoxal	3090715.171 ± 302514.2996	3793133.083 ± 306196.6921	3466899.652 ± 233838.5385	3770208.875 ± 207920.5974	0.18515	0.3703
4-Pyridoxate	199746.6514 ± 55767.95555	158470.47 ± 60056.61754	186436.8607 ± 49068.46936	270994.7433 ± 68436.78006	0.69328	0.8448
Pyridoxamine 5'-phosphate	173421.7406 ± 35819.37564	118431.1495 ± 25964.43649	129494.7682 ± 23558.50971	153914.994 ± 29997.67431	0.65549	0.81174
Nicotinamide	50480857.62 ± 2548591.662	56042133.33 ± 5257155.538	63329871.85 ± 3027571.7	53095851.25 ± 3101652.315	0.054138	0.19532
Nicotinate ribonucleotide	135306.0281 ± 22615.50599	112822.9258 ± 19259.27248	115358.4156 ± 14349.8674	153554.4717 ± 20182.99398	0.80219	0.91274
UDP-glucose	3526732 ± 304651.7253	3151763 ± 215499.8723	3623601.889 ± 243086.1324	2994620.513 ± 215601.2117	0.28021	0.46872
ADP-D-ribose	266607.9152 ± 45756.67573	309583.8875 ± 68306.60652	457932.3 ± 67924.22173	303615.9122 ± 47429.63366	0.1844	0.3703
Phosphate	16893170.86 ± 4998623.651	12907889.5 ± 1684140.941	11322897.63 ± 1188128.635	9610229.208 ± 1134549.407	0.27061	0.45681
D-Glucose	24865903.33 ± 1320068.733	23535771.67 ± 1531129.262	22924651.11 ± 1068218.747	21902862.63 ± 1090601.967	0.40433	0.61485
D-Glyceraldehyde 3-phosphate/Glycerone phosphate	459606.8952 ± 63253.40026	632691.1333 ± 103870.4535	374336.9289 ± 43767.13215	381840.4075 ± 46163.3444	0.13451	0.32142

Pyruvate	2789763.429 ± 181723.4056	2286924.583 ± 145352.6858	2218226.667 ± 119656.3229	2325457.583 ± 162839.8355	0.047958	0.18255
Lactate	149639043.3 ± 9518767.659	144089092.5 ± 12658868.98	150516380.4 ± 9222496.631	148964578.8 ± 11159260.59	0.95967	0.97709
Maltose	527738.5186 ± 131390.4548	1051883.708 ± 168758.5122	1331070.691 ± 186306.0222	1197106.475 ± 127820.2839	0.000604	**0.006169**
Maltotriose	145936.7133 ± 29914.39076	321592.5792 ± 54444.61053	388256.0241 ± 51688.50622	404532.0908 ± 43724.65802	5.94E-05	**0.001093**
Mannitol	474339.9476 ± 109854.8139	864085.35 ± 281461.2253	3540969.636 ± 2926570.651	588424.1075 ± 139829.6526	0.48881	0.70008
D-Ribose	344310.2905 ± 19710.98504	332996.44 ± 31847.02435	342384.777 ± 27431.66462	414365.5158 ± 61346.38856	0.82816	0.91274
D-Rhamnose	520546.371 ± 66531.4363	428314.6333 ± 67026.90095	411465.9222 ± 39482.49324	357882.3938 ± 48450.65482	0.25808	0.4438
D-Arabitol	7685206.619 ± 1273539.019	10917848.67 ± 4225454.514	6678997.63 ± 778706.3473	6119350.75 ± 1271622.315	0.32855	0.53028
Citrate	3961202.943 ± 434648.014	5113611.917 ± 630368.0758	3770243.37 ± 422146.8253	3333745.921 ± 439230.935	0.17394	0.36167
2-Oxoglutarate	426763.9286 ± 31596.17258	444998.1 ± 37526.92863	448827.9481 ± 34685.27861	376320.8675 ± 37014.63149	0.24268	0.43352
2-Oxoglutaramate	97084.10429 ± 7598.290106	93460.8325 ± 5754.614766	81056.20185 ± 4896.172232	76088.59125 ± 4322.567717	0.11693	0.29883
Fumarate	3792296.381 ± 413211.1542	3354768.917 ± 392816.7466	3241774.148 ± 208012.549	3604429.35 ± 314049.1625	0.81826	0.91274
Malate	33844526.67 ± 1941667.832	36123927.5 ± 3500802.572	30039287.04 ± 1828030.24	31682606.29 ± 2640419.375	0.35549	0.56388
Oxaloacetate	79258.69 ± 4300.183238	79904.9625 ± 5434.593827	77378.01148 ± 3690.889961	81206.04208 ± 4444.371539	0.96165	0.97709
Itaconate	1968886.395 ± 186784.9417	2209550 ± 272611.6034	2214100.93 ± 195584.8247	1853393.938 ± 213019.1372	0.36411	0.56777
2-Hydroxyglutarate/Citramalate	217594.471 ± 24577.75475	229240.246 ± 33852.27904	245618.7342 ± 22360.01069	212492.5228 ± 28724.85017	0.64075	0.81174
D-Erythrose 4-phosphate	78560.38276 ± 8737.500476	76054.42833 ± 14437.31791	233381.187 ± 74583.10409	65326.7075 ± 9455.238033	0.18798	0.37192
alpha-D-Ribose 1-phosphate	3226568.367 ± 402911.6634	2062265.858 ± 369331.9816	1909659.919 ± 181645.5738	1940474.513 ± 211190.0963	0.064473	0.21569
Glutathione	20306461.9 ± 1185279.029	16715683.08 ± 2038715.346	19916374.85 ± 1127360.347	16810580.38 ± 1114148.241	0.13897	0.32783
Glutathione disulfide	488054.6048 ± 59423.46124	417906.5917 ± 61150.7524	541505.3296 ± 100257.5302	299437.6942 ± 35815.9711	0.030127	0.14508
5-Oxoproline	13860876.05 ± 557747.7398	17499657.5 ± 1149356.695	15021352.85 ± 703905.6454	11372980.88 ± 629824.0307	8.02E-06	**0.000492**
S-Glutathionyl-L-cysteine	523215.8762 ± 45656.37391	622411.9583 ± 95226.70617	564894.2819 ± 87439.01089	526347.675 ± 61888.14058	0.65421	0.81174
Cys-Gly	315964.6048 ± 22357.50431	344198.2883 ± 61702.09129	296526.5852 ± 20676.14983	307216.2375 ± 23297.75839	0.9437	0.97517
Dehydroascorbate	119658.83 ± 6605.602736	137754.4808 ± 10880.87619	110335.4774 ± 5336.995115	113635.6608 ± 8182.171604	0.12195	0.30737
gamma-L-Glutamyl-D-alanine	155925.091 ± 11659.94986	182477.2583 ± 18014.51716	166287.7581 ± 11228.27778	167853.65 ± 15095.6122	0.75534	0.89282

gamma-L-Glutamylputrescine	674425.7524 ± 70252.33429	985090.875 ± 168417.1544	676145.3148 ± 76122.80281	753741.9917 ± 78173.00028	0.3144	0.51651
(5-L-Glutamyl)-L-glutamine	750602.7762 ± 55296.06833	786175.525 ± 94914.99285	641801.5926 ± 45517.61556	647453.8925 ± 85483.08872	0.23282	0.42414
5-L-Glutamyl-taurine	182661.2067 ± 17411.69209	223746.1408 ± 38201.59646	222215.8074 ± 15110.04275	216282.97 ± 26817.11901	0.49539	0.70008
Cystathionine	50456.85429 ± 10316.15147	127995.5908 ± 24112.95535	133114.5185 ± 19373.27799	143609.4225 ± 20626.36808	0.001973	**0.016498**
Dimethylglycine	5627124.333 ± 776963.407	7160885.583 ± 816220.7745	5334429.667 ± 260124.8767	4749704.113 ± 260335.5867	0.029787	0.14508
Phosphoserine	57536.25048 ± 5453.611194	76278.46833 ± 12784.64118	52803.16963 ± 5732.447203	34506.64458 ± 4425.164278	8.54E-05	**0.00131**
3-Phosphonooxypyruvate	21266270.95 ± 550019.7679	21461585 ± 671841.0893	20599277.78 ± 518505.1463	20661281.25 ± 529408.9578	0.64402	0.81174
S-Adenosyl-L-homocysteine	149564.8619 ± 14793.15007	145916.4358 ± 14917.22617	148784.42 ± 12585.61108	143709.4904 ± 13288.54506	0.97448	0.97981
Ornithine	1390995.195 ± 85235.19856	2005532.5 ± 161085.6968	1559267.963 ± 110110.2102	1297650.933 ± 80576.66259	0.001561	**0.013673**
L-Citrulline	780331.4381 ± 54817.09063	1009893.258 ± 194855.5974	847022.3 ± 72072.42147	845042.3917 ± 86035.78682	0.64179	0.81174
Argininosuccinate	281227.3648 ± 41390.3627	320077.3583 ± 47219.43152	296935.1174 ± 30553.01686	386764.6983 ± 50410.67932	0.75289	0.89282
Putrescine	372896.541 ± 89381.81032	1461170.274 ± 562226.329	377492.0495 ± 73919.98125	172832.8467 ± 36328.48922	0.000585	**0.006169**
Spermidine	736348.6238 ± 102498.3695	2280757.817 ± 586405.9362	3072315.644 ± 899936.078	879878.2458 ± 116499.4862	0.000145	**0.002046**
Spermine	274202.5681 ± 90966.0859	221834.7933 ± 53486.88055	2312577.804 ± 959009.5877	472788.0102 ± 104313.6611	0.00636	0.040353
N-Acetylneuraminate	5934561.381 ± 438325.3559	5098631.917 ± 706112.161	4547974.074 ± 451350.6154	3894022.1 ± 450779.9343	0.011718	0.069554
N-Glycoloyl-neuraminate	258346.2619 ± 8312.444965	287411.1083 ± 20695.71642	327383.3741 ± 14150.3411	341310.475 ± 14192.38936	0.000215	**0.002633**
alpha-D-Glucosamine 1-phosphate	251961.3314 ± 26746.21455	297158.6583 ± 40897.6073	248424.9407 ± 26542.07519	175134.0338 ± 13049.65932	0.087791	0.2495
1-4-beta-D-Xylan	253029.9746 ± 70763.30786	300488.8908 ± 93895.68385	192943.9359 ± 26873.74165	113592.4613 ± 7760.437287	0.04498	0.17927
UDP-N-acetyl-D-glucosamine	2643268.667 ± 145344.1803	3059425.417 ± 243238.7653	3295276.889 ± 199705.1225	2814840.542 ± 186023.2396	0.22006	0.409
CMP-N-acetylneuraminate	361167.2762 ± 22101.89981	363875.0583 ± 25712.99859	332150.7111 ± 25378.27061	284962.2504 ± 17903.5883	0.070172	0.22261
Glycolate	1640383.038 ± 186445.2636	2245577.667 ± 263035.7473	1592289.23 ± 188854.4149	1267660.817 ± 182051.6611	0.020827	0.11892
Homocarnosine	57983.59457 ± 16573.73976	67095.1875 ± 10529.46625	60997.47326 ± 8662.224727	62199.529 ± 9222.770641	0.54406	0.75839
Carnosine	114885.0881 ± 10840.62208	150259.1408 ± 25471.65508	141556.8633 ± 13191.00513	140950.3096 ± 16008.68003	0.67161	0.82384
Creatine	470221638.1 ± 34246108.57	386660058.3 ± 31146226.32	435472037 ± 28672902.4	324129883.3 ± 26325848.8	0.002427	**0.019416**
Creatinine	9858506.333 ± 457245.9966	9631036.417 ± 646704.5079	10140939.44 ± 454253.9532	9653820.167 ± 513988.4917	0.82728	0.91274

4-Acetamidobutanoate	470137.9571 ± 25325.92097	576056 ± 115330.4287	451164.1593 ± 13409.77624	433188.9542 ± 12526.20907	0.155	0.34689
N-Acetylornithine	279527.4176 ± 40653.98811	453999.425 ± 100741.2104	274128.3089 ± 22994.85097	289619.5033 ± 19829.93655	0.041696	0.17163
Guanidinoacetate	1424898.024 ± 137202.9136	1497117.517 ± 184161.2223	1771765.211 ± 139301.2117	1379835.025 ± 127911.1563	0.15783	0.34689
Pantothenol	1768216.729 ± 231540.6989	2560883.483 ± 642992.3581	2983151.7 ± 300321.0782	3077703.263 ± 436163.1937	0.035412	0.15892
Pantetheine	23420.44324 ± 4568.932499	28249.77333 ± 5759.689949	20438.64104 ± 2877.53873	22406.72458 ± 4117.682027	0.64247	0.81174
Taurine	75061032.86 ± 2141985.722	71672602.5 ± 6533569.581	71317302.59 ± 3649912.172	64253597.92 ± 3653198.844	0.19996	0.38728
3-Sulfino-L-alanine	403152.4186 ± 57409.46145	508739.575 ± 90441.72975	473547.0852 ± 50843.80832	483424.9 ± 51052.77481	0.56466	0.77463
L-Cysteate	138167.2362 ± 29271.65533	174639.0863 ± 50106.56301	155919.0536 ± 28148.84322	172045.9604 ± 26731.66211	0.9123	0.97038
L-Methionine S-oxide	384735.0048 ± 25344.24285	540930.5917 ± 86393.4108	437368.6663 ± 35388.12869	497050.1304 ± 40161.29442	0.64393	0.81174
5-Hydroxyindoleacetate	829192.7762 ± 89758.06848	933906.5583 ± 188686.2296	938430.2778 ± 87909.12851	883392.475 ± 98266.35678	0.79712	0.91274
Indole	259131.6495 ± 22691.0187	326521.3 ± 35311.34634	362288.4778 ± 28116.86577	344405.6683 ± 29943.76729	0.057002	0.19789
Indole-3-acetaldehyde	2615993.876 ± 412856.2642	2230864.817 ± 421247.8144	1537196.783 ± 252568.4011	1069376.325 ± 236610.6076	0.000995	**0.009638**
Indole-3-acetate	153534.1238 ± 34121.51992	180592.8933 ± 49665.53922	69601.36274 ± 12671.46535	36118.72042 ± 6075.036602	0.000188	**0.002473**
6-Hydroxykynurenic acid	153484.6119 ± 27519.93806	291204.415 ± 52203.76785	127897.437 ± 17313.78031	139338.1571 ± 23367.25968	0.10631	0.2835
kynurenine	2000662.024 ± 195528.9873	3977533.45 ± 824108.6734	3299929.789 ± 435845.5323	2826344.221 ± 355516.2343	0.097916	0.2689
Anthranilate	158519.2338 ± 31569.72288	333293.3875 ± 193137.1958	178384.3267 ± 42676.32338	152550.7179 ± 43994.41134	0.58206	0.78749
Picolinic acid	498775.4124 ± 136357.1721	782338.085 ± 581125.2687	337491.0785 ± 124919.4734	167731.9279 ± 43644.30312	0.067055	0.21646
g-Oxalo-crotonate	10201091.9 ± 690872.1046	11179471.08 ± 1291616.48	9261336.704 ± 580834.0272	10838172.04 ± 890360.7194	0.43279	0.64743
2-Oxoadipate	306617.9048 ± 13449.90599	256120.8333 ± 10880.00776	292064.5852 ± 14440.24256	291985.65 ± 12284.77864	0.25217	0.4397
L-Adrenaline	792259.1905 ± 64406.9368	1213345.092 ± 321986.8369	872973.9333 ± 33803.16939	884243.8 ± 97050.1861	0.17693	0.36173
Serotonin	2422737.895 ± 372982.9069	2084072.5 ± 376084.9241	1404719.469 ± 234471.4455	1038908.982 ± 222836.1206	0.002898	**0.022215**
N-Methylethanolamine phosphate	4137902.619 ± 372068.0305	4896688.75 ± 697252.9013	3863772.519 ± 296452.7251	3026063.317 ± 384725.4438	0.003433	**0.024293**
Sphingosine	177867.5729 ± 20489.32226	231552.4833 ± 40916.76024	331240.9 ± 117134.5833	671249 ± 496376.7511	0.49695	0.70008
Sphingosine 1-phosphate	438705.2095 ± 18353.17297	486376.1917 ± 35496.25875	557613.5963 ± 34440.40331	537803.95 ± 41748.89698	0.13421	0.32142
sn-glycero-3-Phosphoethanolamine	7129362.905 ± 385867.3477	5094260.583 ± 576893.3578	7405221.889 ± 407514.4195	6699093.458 ± 413442.3862	0.021329	0.11892

CDP-choline	122236.6471 ± 11446.94809	132473.6367 ± 10583.11596	170509.477 ± 14432.55529	153826.8017 ± 15007.45442	0.14206	0.33088
Choline	379132.1524 ± 29856.82765	442557.3 ± 35156.28157	437726.0926 ± 19020.52308	420171.575 ± 24569.14949	0.21753	0.409
Sphinganine 1-phosphate	440689.4 ± 25879.91872	610658.2583 ± 149628.9786	443462.8148 ± 36326.38972	434881.7167 ± 35122.78435	0.35999	0.56614
L-Carnitine	46440858.1 ± 2751783.152	49959956.67 ± 3240583.184	55006864.07 ± 2478711.071	45449189.17 ± 2584547.321	0.045792	0.17927
acyl-C2 (acetyl-carnitine)	1992100.229 ± 249817.9859	1980514.458 ± 145743.4831	1730656.833 ± 172666.0706	1487057.983 ± 175102.9669	0.13096	0.32142
acyl-C3 (propionyl-carnitine)	6386862.681 ± 1386124.59	6877513.5 ± 1687920.654	4894218.159 ± 674982.3071	2956770.75 ± 389219.1214	0.056256	0.19789
acyl-C4 (butanoyl-l-carnitine)	2909004.486 ± 601470.2455	2704416.625 ± 550663.9455	1988869.078 ± 294363.1372	1831215.283 ± 526732.7812	0.1708	0.36124
acyl-C4-OH (Hydroxybutyrylcarnitine)	599150.2524 ± 72033.84841	686370.3083 ± 89342.82664	676999.3489 ± 95308.95728	818103.6538 ± 108207.9516	0.59271	0.79028
acyl-C5-OH	135666.8729 ± 20928.05905	267045.7492 ± 107151.1295	236164.1811 ± 63556.60251	119166.1188 ± 15318.42366	0.21865	0.409
acyl-C6 (hexanoyl-l-carnitine)	350952.8571 ± 23414.44737	387466.175 ± 34142.26219	465800.3148 ± 151246.7682	377778.275 ± 31271.01412	0.91764	0.97038
acyl-C8 (L-octanoylcarnitine	223913.1952 ± 22081.62343	206500.9167 ± 17235.95612	224682.237 ± 64869.18465	200203.6267 ± 19532.85443	0.47038	0.68691
acyl-C8:1 (octenoyl-l-carnitine)	303427.6524 ± 34111.34913	285355.6508 ± 50753.92828	299552.8644 ± 47028.43975	236226.9304 ± 24825.3547	0.55719	0.77085
acyl-C10 (O-Decanoyl-L-carnitine)	103859.841 ± 23341.00055	71608.25833 ± 9861.327762	66919.0537 ± 9563.836126	56750.36833 ± 13862.54514	0.027157	0.14277
acyl-C12 (O-dodecanoyl-carnitine)	78888.57781 ± 21921.78591	89638.61817 ± 20896.02828	78169.75889 ± 10248.41801	43349.57417 ± 9278.247953	0.093023	0.25934
acyl-C14:1 (Tetradecenoyl Carnitine)	241568.6476 ± 20168.10223	200243.7083 ± 11040.89841	205967.4296 ± 15519.58591	204608.1458 ± 10217.13822	0.26272	0.44759
acyl-C16:1 (Hexadecenoyl-carnitine)	219603.5219 ± 55306.81023	191835.2217 ± 36703.83939	215704.6567 ± 35540.59797	126867.9246 ± 24019.93418	0.65266	0.81174
Butanoic acid	290100.1505 ± 105070.0501	245240.9958 ± 66462.49489	185960.657 ± 25559.20906	163575.5358 ± 17650.83141	0.2533	0.4397
Hexanoic acid (caproate)	3143597.143 ± 215585.1811	3309715.333 ± 279699.5275	3728777.481 ± 857518.9072	3234808.208 ± 276686.8179	0.96647	0.97709
Heptanoic acid	1981717.714 ± 78031.93237	2263975.917 ± 181779.656	2489711.593 ± 652120.6966	2060139.683 ± 134674.6637	0.81803	0.91274
Octanoic acid (caprylate)	8706687.952 ± 387940.494	8405575.083 ± 723567.8998	11194730.74 ± 3065424.256	9751068.125 ± 646440.6744	0.80066	0.91274
Nonanoic acid (pelargonate)	53888140.48 ± 2332651.66	56390653.33 ± 5613217.577	69881178.52 ± 21726562.62	61436228.33 ± 5350180.661	0.91548	0.97038
Decanoic acid (caprate)	16544701.43 ± 642224.9253	15642307.58 ± 1332263.375	20538967.33 ± 6108167.949	16915474.88 ± 1159765.838	0.93547	0.97517
Dodecanoic acid	11274747.81 ± 599363.7212	12461668.83 ± 2109214.61	12777554.59 ± 3221402.001	10952468.75 ± 691148.0671	0.80755	0.91274
Tetradecanoic acid	13588589.57 ± 581324.9551	23579256.25 ± 10773396.17	15130624.96 ± 3715202.018	11868389.63 ± 665662.907	0.28875	0.47865

Hexadecanoic acid	101125578.6 ± 4360131.269	147363645 ± 28899883.37	109737968.5 ± 9883392.928	102886157.5 ± 5967210.173	0.065876	0.21645
Octadecanoic acid	60912732.86 ± 2966439.633	109166493.3 ± 40022669.69	64357890.74 ± 4741999.397	60695962.5 ± 3056465.959	0.075874	0.23268
Tetradecenoic acid	879851.6524 ± 53993.48337	1037644.117 ± 126459.5606	1539632.348 ± 587804.7915	897853.2667 ± 123521.934	0.56834	0.77463
Hexadecenoic acid	11981189.05 ± 958734.7239	16049798.17 ± 2001733.358	19633995.63 ± 5599802.103	12598226.75 ± 1980980.837	0.11604	0.29883
Octadecenoic acid	111217934.8 ± 5809582.263	140585695.8 ± 11746410.34	149228675.2 ± 9218352.933	143931121.3 ± 12975145.71	0.041975	0.17163
Linoleate	116868305.2 ± 6001755.826	165576694.2 ± 19727327.21	169986951.5 ± 15350380.05	177246671.3 ± 18914745.72	0.051469	0.18941
Octadecatrienoic acid	4523766.286 ± 377188.2469	4755175.917 ± 490249.2294	4845440.111 ± 408211.1424	5200188.375 ± 1282889.133	0.88293	0.95564
Eicosatetraenoic acid	95971517.14 ± 7137815.839	158062830.8 ± 25296693.79	181546588.9 ± 16226105.18	176679990.4 ± 19412360.49	0.000363	**0.004175**
Eicosapentaenoic acid	6699400.524 ± 611515.0981	11736396.83 ± 2751997.578	16767298.81 ± 2322643.861	17570809.38 ± 2354124.929	2.54E-05	**0.000667**
Docosahexaenoic acid	8115050.762 ± 721090.0456	19086451.58 ± 4078823.188	26144847.04 ± 2936717.292	26480725.71 ± 4455222.564	2.20E-07	**2.02E-05**
Dodecanedioic acid	139751.5848 ± 6467.229436	135412.2142 ± 15410.72535	184780.2681 ± 55708.38756	146815.4538 ± 11999.98875	0.94867	0.97517
(8Z-11Z-14Z)-Icosatrienoic acid	14193957.43 ± 1801555.278	19785411 ± 3362351.274	23889836.96 ± 2205056.83	17869849.42 ± 1723807.897	0.003645	**0.024842**
(5Z-8Z-11Z-14Z-17Z)-Icosapentaenoic acid	6699400.524 ± 611515.0981	11736396.83 ± 2751997.578	16767298.81 ± 2322643.861	17570809.38 ± 2354124.929	2.54E-05	**0.000667**
(7Z-10Z-13Z-16Z-19Z)-Docosa-7-10-13-16-19-pentaenoic acid	4038156.81 ± 293169.0534	9299141.333 ± 2105724.894	10112845.07 ± 919187.8795	9746609.125 ± 1409021.949	1.37E-05	**0.000503**
Bilirubin	228335.661 ± 42599.3384599999	353123.8067 ± 73887.33059	557972.3741 ± 85619.69757	659352.1896 ± 109163.9224	5.23E-05	**0.001069**
Triacanthine	20451582 ± 2568812.176	20533485.83 ± 1487866.454	18059756.56 ± 1710684.324	15237731.21 ± 1709934.77	0.13283	0.32142
N5-Methyl-L-glutamine	68051.66048 ± 8968.960862	90834.75667 ± 17067.91469	67644.90926 ± 9912.692079	59244.82208 ± 7621.255233	0.39679	0.61352
2-Methyleneglutarate	314972.84 ± 45397.92874	282858.675 ± 36989.01573	335456.3693 ± 37568.07905	376346.1458 ± 43926.49701	0.60733	0.80395
beta-Butoxyethyl nicotinate	463913.4571 ± 10035.77812	469203.1833 ± 11957.81726	500895.9333 ± 45525.04484	453989.1542 ± 8014.677716	0.74796	0.89282
2-Deoxy-alpha-D-glucoside	9963803.619 ± 893902.285	14308468.75 ± 1722618.895	14884634.89 ± 2545391.786	10032889.25 ± 864056.9824	0.026689	0.14277
cis-p-Coumarate	640390.2619 ± 38104.72966	824820.7167 ± 77993.66965	814516.5778 ± 61585.08411	904235.2542 ± 75932.10899	0.063966	0.21569
N6-Methyl-L-lysine	789946.5952 ± 128570.0569	730525.3667 ± 103447.9621	647860.3593 ± 87983.1039	686449.9542 ± 86019.82642	0.75695	0.89282
D-glucono-1,5-lactone	363593.6238 ± 24490.6264	338331.9167 ± 24019.87183	348041.1222 ± 14980.37799	330870.3417 ± 17536.15041	0.65734	0.81174
Dihydroflavonol	32017693.33 ± 1585077.846	27701040.83 ± 1845301.081	30595482.59 ± 1434617.744	29141882.79 ± 1447411.051	0.43942	0.65204
Glucosinolate	184828.7962 ± 25950.53452	162821.3192 ± 19223.69062	180362.0174 ± 31864.19367	209264.8588 ± 23536.3865	0.89589	0.964

Thioredoxin disulfide	137189.0476 ± 25914.8679	227347.8433 ± 39566.09331	217158.7311 ± 55669.84386	123882.2517 ± 19028.43889	0.081054	0.24055
Pyridoxamine	630867.6476 ± 42137.89223	748182.75 ± 90567.77012	652905.1185 ± 41083.08453	817524.3583 ± 56669.90191	0.08814	0.2495
9-Oxononanoic acid	1154036.252 ± 56196.52339	1155656.808 ± 105425.5562	1205290.07 ± 199549.4615	1126542.854 ± 76505.3528	0.85594	0.93578
N-Amidino-L-aspartate	61247.69952 ± 14659.15982	112333.3545 ± 34921.84687	42065.69548 ± 9720.638507	21185.45325 ± 4658.675183	0.15836	0.34689
Glycerone sulfate	62409.54143 ± 15744.38691	43844.7925 ± 8209.663863	45437.80074 ± 5584.624136	47739.12583 ± 5827.110795	0.85949	0.93578
N-Acyl-D-aspartate	8824287.524 ± 729017.3891	11792754.75 ± 1201918.306	11098316.89 ± 995457.2907	8735630.75 ± 614101.7821	0.03777	0.16547
6-Carboxyhexanoate	68754.78524 ± 11412.23884	104149.1867 ± 38954.99408	155533.3485 ± 101346.4348	97262.82208 ± 29193.5360199999	0.40428	0.61485
N-Acetylmethionine	515321.6581 ± 75198.02214	631498.35 ± 65103.13793	521300.0296 ± 46702.39083	545449.4725 ± 67581.94072	0.42387	0.63928
10-Hydroxydecanoic acid	336748.1048 ± 15558.8578	376611.0667 ± 54003.86232	338648.1963 ± 78532.84415	300690.85 ± 25101.70381	0.1965	0.38465
Oxalosuccinate	240967.9048 ± 16223.31761	415929.9917 ± 138474.5925	269562.5389 ± 18948.42319	231301.6821 ± 17899.56891	0.048614	0.18255
2-Oxo-7-methylthioheptanoic acid	1069646.929 ± 62357.81062	1226759.8 ± 122158.6845	1069788.363 ± 72987.53189	1490944.017 ± 109343.5587	0.008549	0.052436
Homomethionine	73297.22352 ± 11847.49743	115989.9867 ± 13944.55282	93760.47719 ± 8926.489497	88577.48225 ± 13395.53181	0.034111	0.15691

Ultra high pressure liquid chromatography-mass spectrometry-based high throughput metabolomics was performed and identified a total of 184 named metabolites demonstrated above. Relative tissue concentration ± SEM reported based upon identical initial tissue weight. ANOVA P value and FDR P value noted as above, with significant values of P<0.05 highlighted in bold.

Supplemental Figure 2

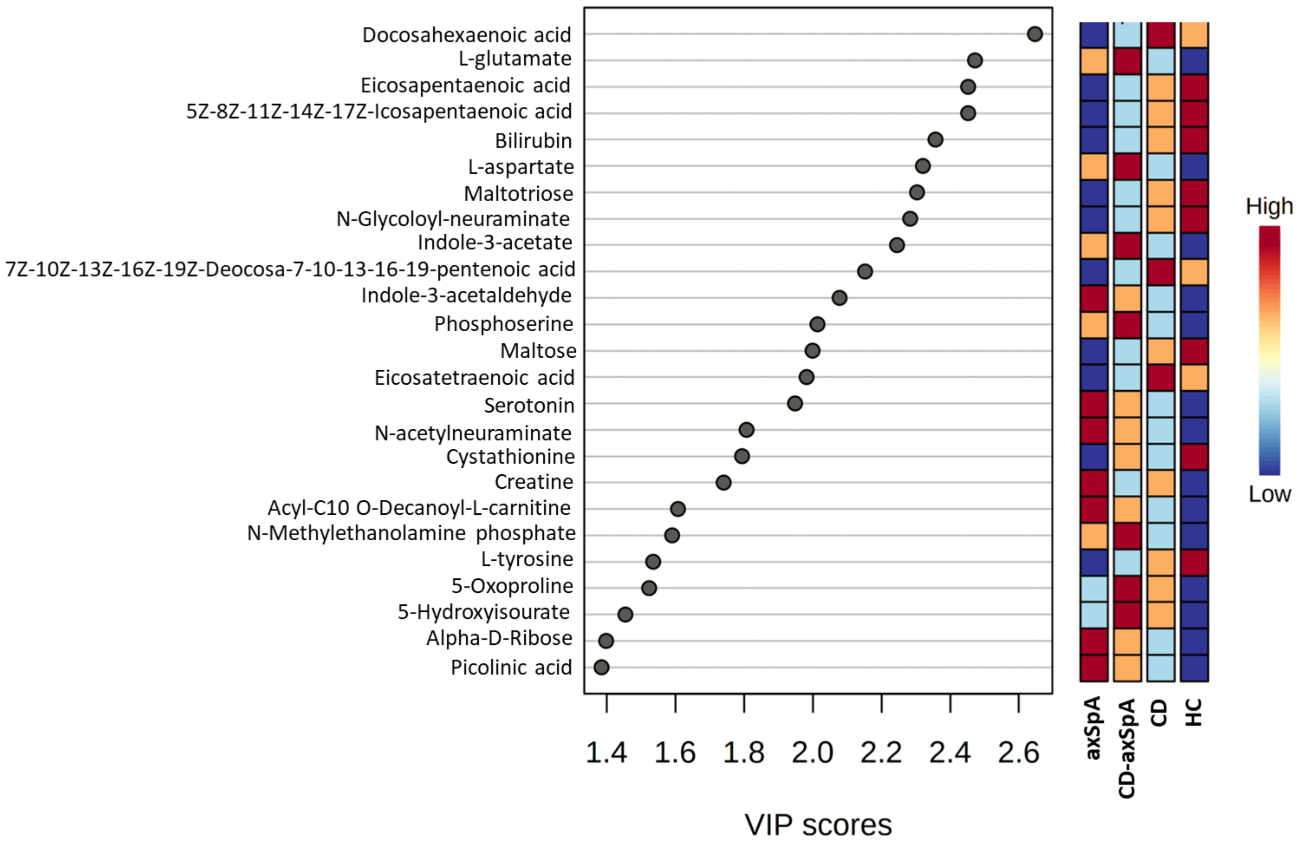

Supplemental Figure 2. Top 25 significant metabolic drivers of differences across disease states demonstrated as VIP plot. Biopsy samples from groups HC, axSpA, CD, and CD-axSpA were evaluated by LC-MS detection of 184 metabolites. The top 25 metabolites that are driving the separation of all groups in PLSDA analysis are shown.

Supplemental Figure 3

A)

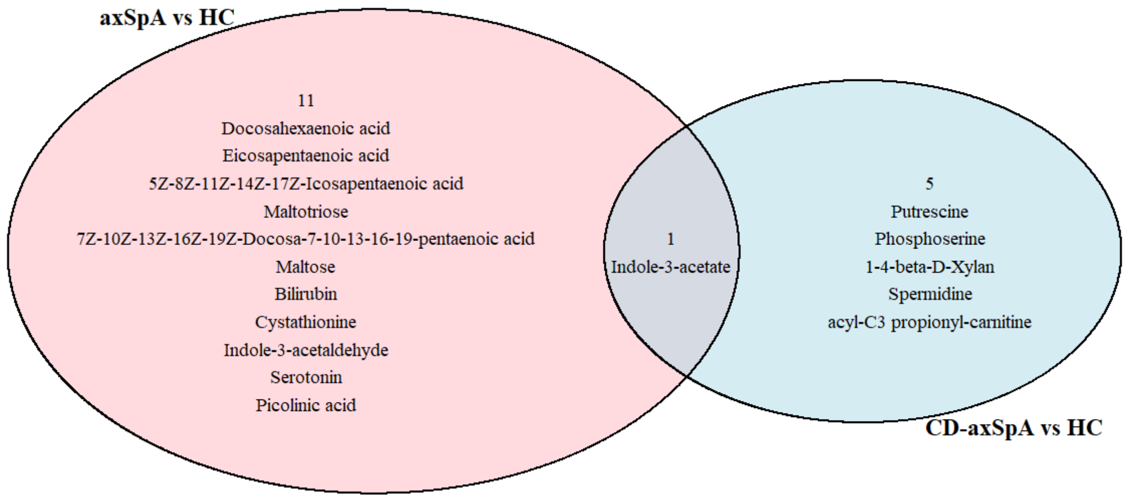

B)

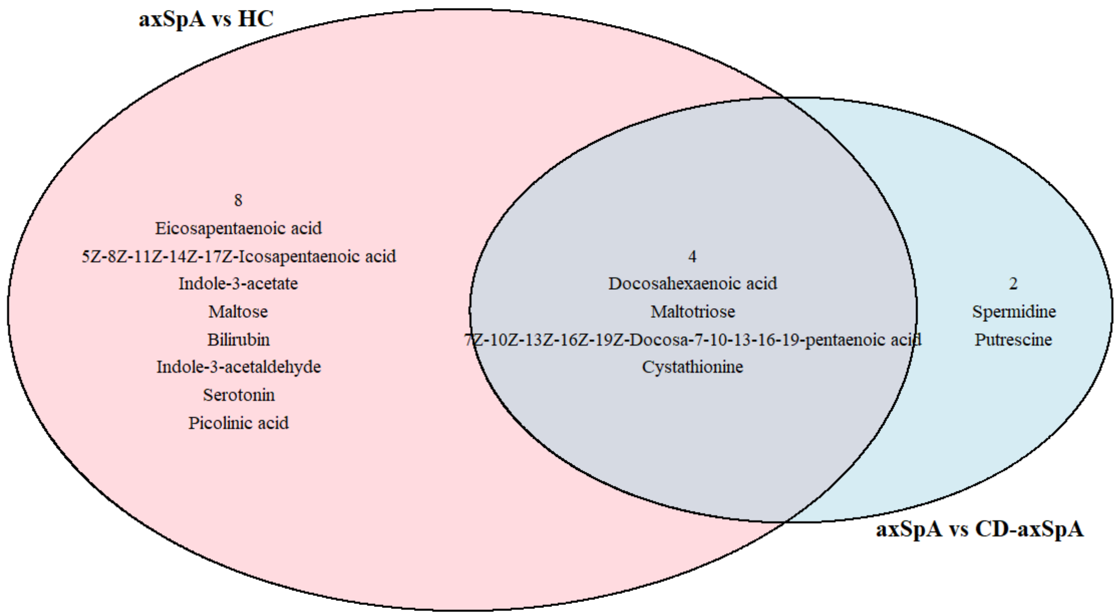

C)

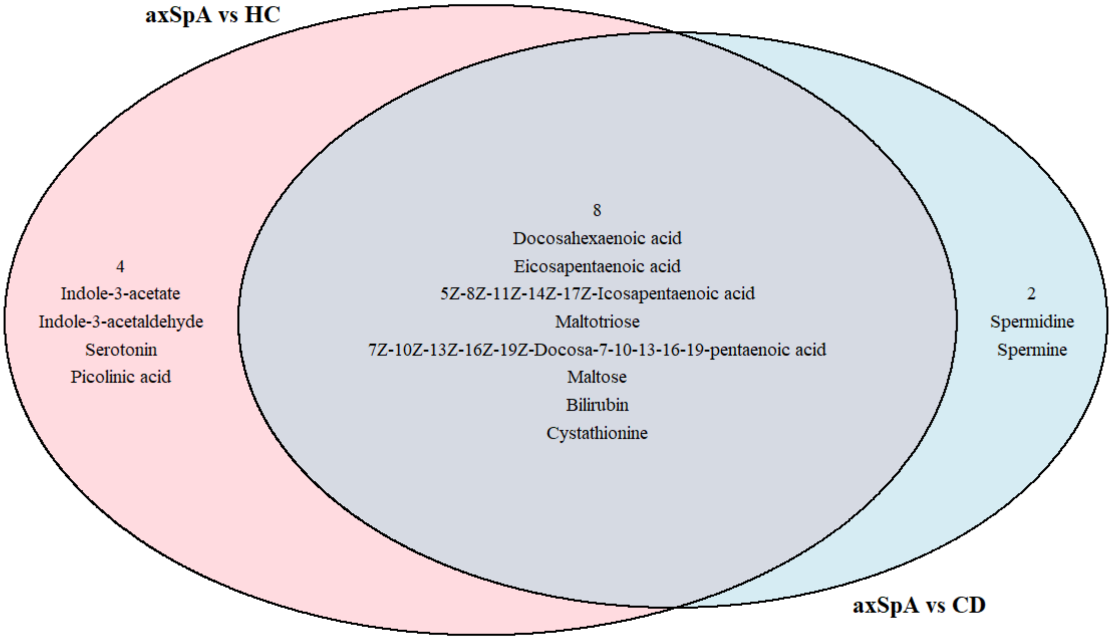

Supplemental Figure 3. Venn diagrams demonstrating overlapping significant pairwise differences. Pairwise comparisons between groups within each labeled circle were performed. In the circles are the metabolites meeting $p<0.05$ with post FDR correction of 0.05 and a 2-fold change in the pairwise comparison. Venn diagrams demonstrate which metabolites do and do not overlap in the pairwise comparisons. Comparisons between CD vs HC and CD-axSpA vs CD had no significant hits post FDR.

Supplemental Table 3. Metagenomic paired-end read counts across disease states.

Group	Raw paired-end reads	Paired-end reads following quality control
axSpA	7,874,610±1,765,741	1,448,835±2,043,378
CD	23,458,021±9,039,626	1,822,197±2,069,588
CD-axSpA	23,982,607±7,473,020	2,291,875±2,184,367
HC	8,487,020±874,485	1,121,841±1,276,699

Metagenomic shotgun sequencing performing on NovaSeq6000 platform of 2x150 base pair paired end reads. Raw data processed through FastQC v0.11.9, then concatenated, and analyzed in Kneaddata 0.7.5 for filtering prior to HUMAnN 2.0 pipeline.

Supplemental Figure 4

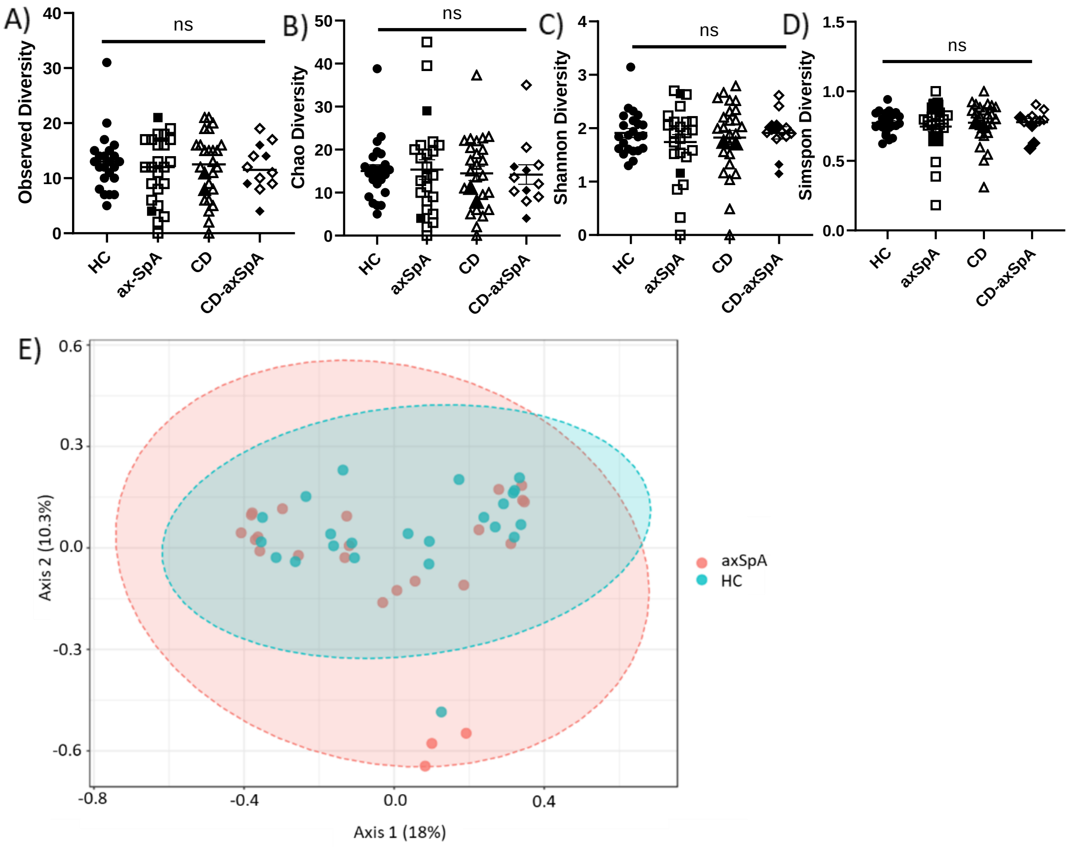

Supplemental Figure 4. Alpha and beta diversity are not significantly different between axSpA and HC. Alpha diversity calculated in MicrobiomeAnalyst using the methods of (A) Observed, (B) Chao1, (C) Shannon, and (D) Simpson. Values for each subject are shown as a symbol, with closed symbols indicating the presence of TNFi use. Bars represent the group means ± SEM. No significant differences between groups was determined by ANOVA. (E) Beta diversity by PCA demonstrates no difference between axSpA and HC groups in regards to species level differences between HC and axSpA.

Supplemental Figure 5

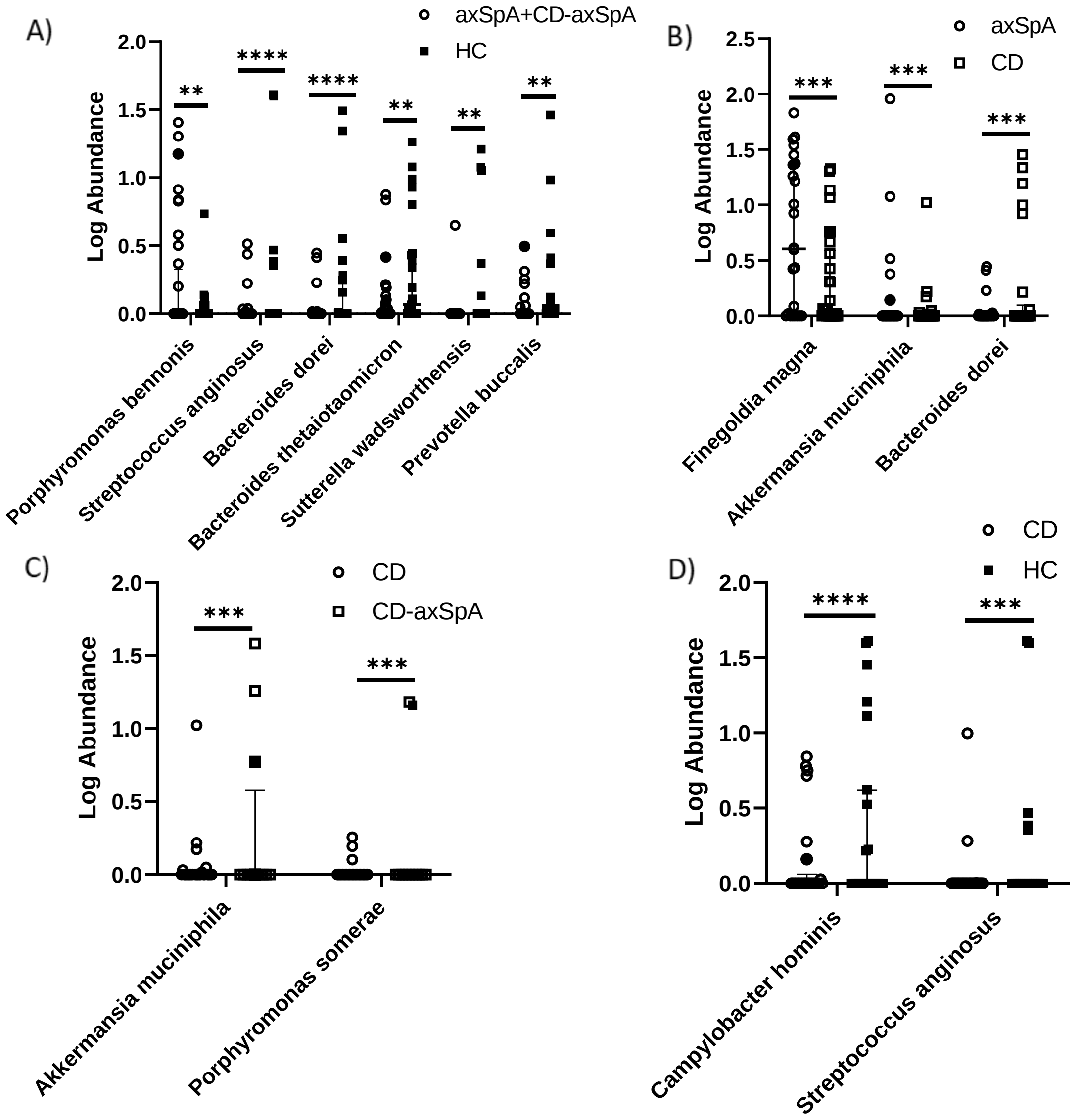

Supplemental Figure 5. Comparisons between subject groups on a species level reveal shifts in specific taxa. axSpA group was combined with CD-axSpA and compared with HC (A), noting a total of 6 species level differences. axSpA versus CD demonstrates three different species (B). Comparison of CD versus CD-axSpA (C) identifies two different species. CD versus HC demonstrates two different species. Data are shown as symbols for the log abundance of the taxa for each subject, with closed symbols representing those taking TNFi, and bars as the group median ± IQR. ** $p<0.005$, *** $p<0.0005$, **** $p<0.00005$.

Supplemental Table 4. Mean log abundance of taxonomic profiling across disease states.

axSpA vs HC	axSpA Median/Mean	HC Median/Mean	p-value	FDR
Porphyromonas bennonis	0/0.250	0/0.056	0.001	0.055
Bifidobacterium adolescentis	0/0.203	0/0.030	7.22×10^{-4}	0.055
Streptococcus anginosus	0/0.034	0/0.192	1.76×10^{-4}	0.028
Bacteroides dorei	0/0.047	0/0.195	0.001	0.055
**axSpA vs CD-axSpA**	**axSpA Median/Mean**	**CD-axSpA Median/Mean**	**p-value**	**FDR**
Prevotella bivia	0/0.059	0/0.145	3.0×10^{-4}	0.051
**axSpA+CD-axSpA vs HC**	**axSpA+CD Median/Mean**	**axSpA Median/Mean**	**p-value**	**FDR**
Porphyromonas bennonis	0/0.225	0/0.056	0.002	0.072
Streptococcus anginosus	0/0.035	0/0.192	5.12×10^{-6}	9.59×10^{-4}
Prevotella buccalis	0/0.056	0/0.180	0.002	0.072
Bacteroides thetaiotamicron	0/0.088	0/0.209	0.001	0.066
Sutterella wadsworthensis	0/0.018	0/0.167	0.002	0.072
Bacteroides dorei	0/0.032	0/0.195	9.11×10^{-5}	0.008
**axSpA vs CD**	**axSpA Median/Mean**	**CD Median/Mean**	**p-value**	**FDR**
Finegoldia magna	0.6040.723	0.0580.667	3.30×10^{-4}	0.021
Akkermansia muciniphila	0/0.170	0/0.058	3.48×10^{-4}	0.021
Bacteroides dorei	0/0.047	0/0.238	1.88×10^{-4}	0.021
**CD vs CD-axSpA**	**CD Median/Mean**	**CD-axSpA Median/Mean**	**p-value**	**FDR**
Akkermansia muciniphila	0/0.058	0/0.301	1.15×10^{-4}	0.019
Porphyromonas somerae	0/0.021	0/0.195	8.97×10^{-4}	0.077
**CD vs HC**	**CD Median/Mean**	**HC Median/Mean**	**p-value**	**FDR**
Campylobacter hominis	0/0.137	0/0.283	8.88×10^{-5}	0.014
Streptococcus anginosus	0/0.049	0/0.201	7.49×10^{-4}	0.058

Data from metagenomics taxonomic profiling analysis performed using the EdgeR feature of MicrobiomeAnalyst with a significance cutoff of $p<0.05$ and FDR<0.1. Mean log median and mean abundance presented across disease states.

Supplemental Figure 6

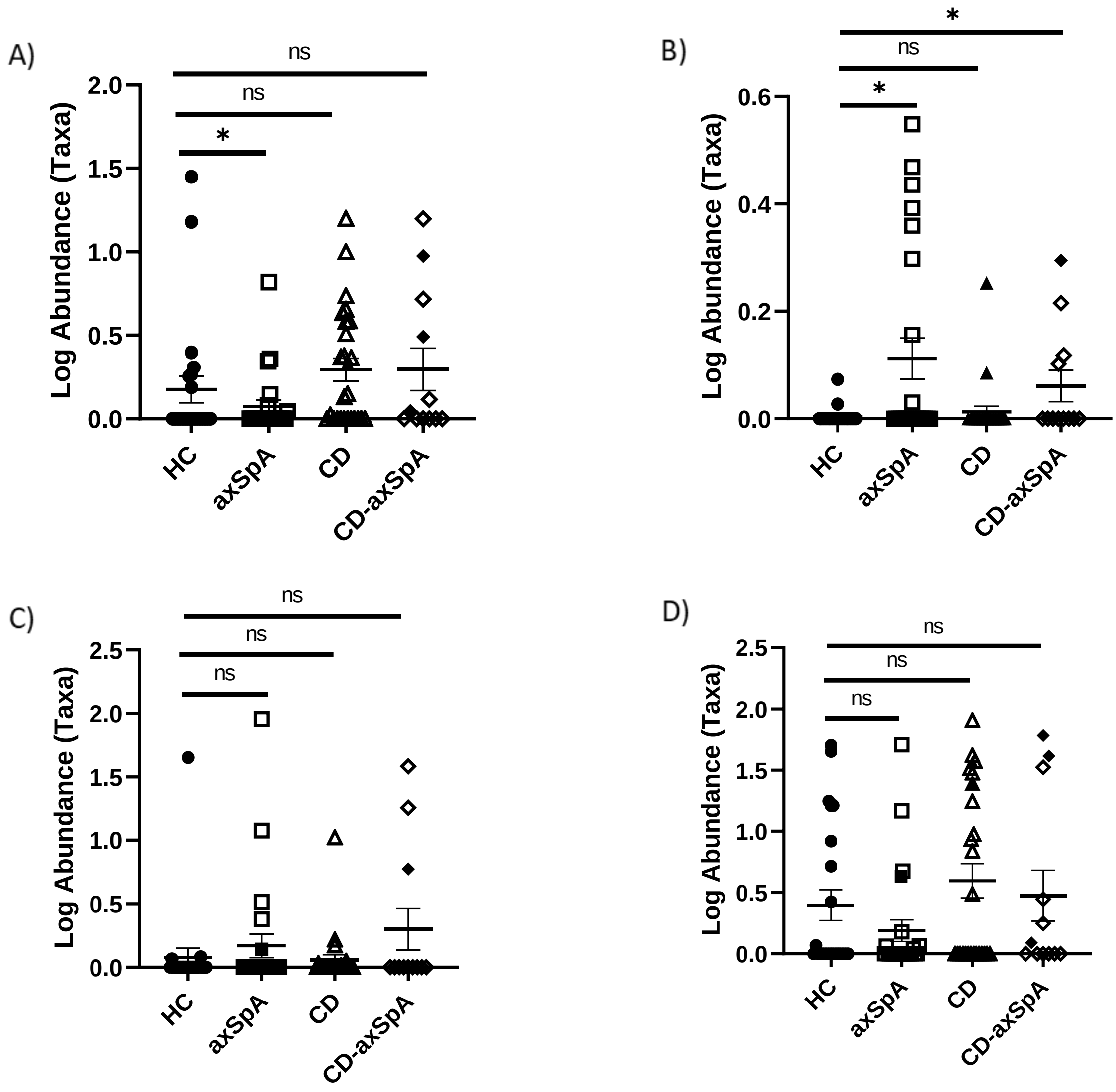

Supplemental Figure 6. Comparison of relative abundances of previously published disease-relevant species across study subject groups demonstrates *Dialister invisus* is expanded in axSpA. The relative abundance of previously published disease-relevant taxa including (A) *Ruminococcus gnavus*, (B) *Dialister invisus*, (C) *Akkermansia muciniphila*, and (D) *Escherichia coli* are compared across all four subject groups. Data are shown as symbols for the log abundance of the taxa for each subject, with closed symbols representing those taking TNFi, and bars as the group mean ± SEM. Statistical significance was determined by ANOVA with Kruskal-Wallis post-hoc test. Ns, not significant; * $p<0.05$.

Supplemental Figure 7

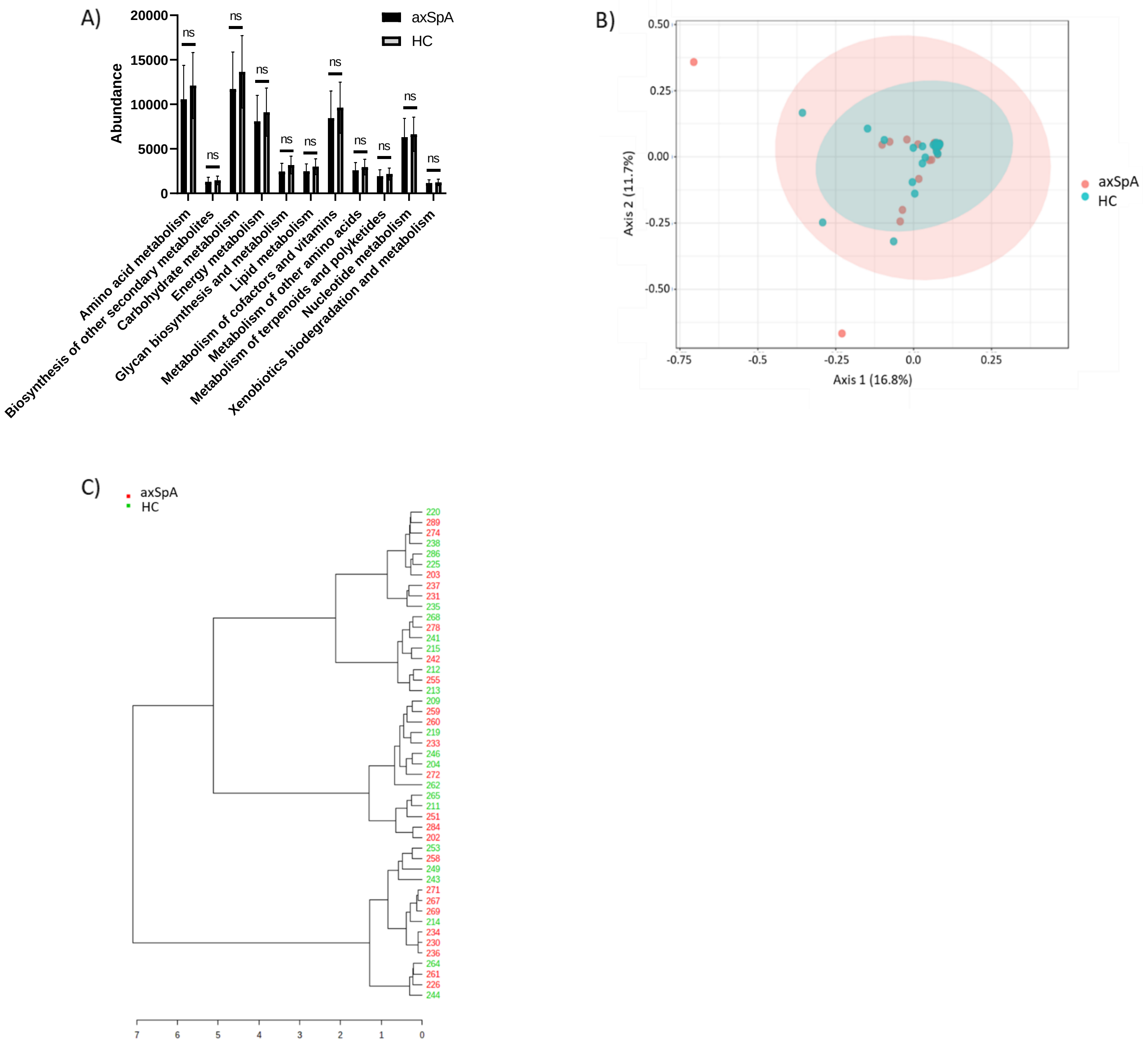

Supplemental Figure 7. KEGG pathway analysis of metagenomic data from subjects with axSpA compared to HC does not reveal significantly altered pathways. (A) Diversity analysis of KEGG metabolic pathways by metabolic category demonstrates no difference in pathways between axSpA and HC. Data represents mean ± SEM. (B) PCA of axSpA versus HC for unbiased metagenomic KEGG data demonstrates no difference between groups. (C) Dendrogram demonstrating separation between axSpA and HC based upon metagenomic data Demonstrates no clear separation. Ns, not significant.

Supplemental Table 5. HUMAnN 2.0 pathway analysis comparing axSpA vs HC.

Pathway	log_2FC	P-value	FDR
PWY-922: mevalonate pathway I	-3.8565	3.01E-07	4.71E-05
PWY66-389: phytol degradation	-3.8166	7.68E-07	7.01E-05
PWY-7446: sulfoglycolysis	3.1645	7.83E-07	7.01E-05
GLUCARDEG-PWY: D-glucarate degradation I	2.9316	2.20E-06	0.000107
PWY1G-0: mycothiol biosynthesis	-3.0426	2.47E-06	0.000112
ARGDEG-PWY: superpathway of L-arginine, putrescine, and 4-aminobutanoate degradation	2.4162	1.48E-05	0.000402
ORNARGDEG-PWY: superpathway of L-arginine and L-ornithine degradation	2.4162	1.48E-05	0.000402
PWY66-398: TCA cycle III (animals)	-2.5413	1.77E-05	0.000442
GALACTARDEG-PWY: D-galactarate degradation I	2.5953	1.93E-05	0.000468
GLUCARGALACTSUPER-PWY: superpathway of D-glucarate and D-galactarate degradation	2.5953	1.93E-05	0.000468
PWY-5005: biotin biosynthesis II	-2.1188	2.25E-05	0.000536
PWY-5845: superpathway of menaquinol-9 biosynthesis	2.7679	3.61E-05	0.000761
PWY-5850: superpathway of menaquinol-6 biosynthesis I	2.7679	3.61E-05	0.000761
PWY-5896: superpathway of menaquinol-10 biosynthesis	2.7679	3.62E-05	0.000761
ORNDEG-PWY: superpathway of ornithine degradation	2.2243	3.75E-05	0.000776
PWY-5860: superpathway of demethylmenaquinol-6 biosynthesis I	2.5589	4.92E-05	0.000966
PWY-5862: superpathway of demethylmenaquinol-9 biosynthesis	2.5589	4.93E-05	0.000966
PWY0-321: phenylacetate degradation I (aerobic)	2.0804	7.44E-05	0.001389
PWY-4041: γ-glutamyl cycle	2.7219	7.62E-05	0.001412
PWY-6823: molybdenum cofactor biosynthesis	2.3208	8.96E-05	0.001624
PWY0-1338: polymyxin resistance	2.1539	9.79E-05	0.001716
UBISYN-PWY: superpathway of ubiquinol-8 biosynthesis (prokaryotic)	2.2471	0.000239	0.003152
PWY-5138: unsaturated, even numbered fatty acid β-oxidation	1.7373	0.000322	0.003838
ECASYN-PWY: enterobacterial common antigen biosynthesis	2.2162	0.000379	0.004245
PWY-6895: superpathway of thiamin diphosphate biosynthesis II	2.5213	0.000415	0.00447
PWY-6803: phosphatidylcholine acyl editing	2.2995	0.000587	0.005461
LPSSYN-PWY: superpathway of lipopolysaccharide biosynthesis	2.1185	0.000608	0.005589
PWY-7315: dTDP-N-acetylthomosamine biosynthesis	2.2714	0.000698	0.006128
PWY-5855: ubiquinol-7 biosynthesis (prokaryotic)	2.046	0.00074	0.006192
PWY-5856: ubiquinol-9 biosynthesis (prokaryotic)	2.046	0.000741	0.006192
PWY-5857: ubiquinol-10 biosynthesis (prokaryotic)	2.046	0.000741	0.006192
PWY-6708: ubiquinol-8 biosynthesis (prokaryotic)	2.046	0.000742	0.006192
PWY-6891: thiazole biosynthesis II (Bacillus)	2.0985	0.000807	0.006477
PWY-5509: adenosylcobalamin biosynthesis from cobyrinate a,c-diamide I	-1.707	0.00086	0.006703
PWY0-1533: methylphosphonate degradation I	2.1111	0.000979	0.007385
PWY-4702: phytate degradation I	2.0961	0.001008	0.007551
PWY-7204: pyridoxal 5'-phosphate salvage II (plants)	2.0665	0.001252	0.008867
PWY-5723: Rubisco shunt	2.1724	0.001498	0.010005
PWY-5861: superpathway of demethylmenaquinol-8 biosynthesis	2.103	0.002056	0.012391
**PWY-6629: superpathway of L-tryptophan biosynthesis**	**2.1065**	**0.002069**	**0.012391**
PWY-5838: superpathway of menaquinol-8 biosynthesis I	2.1407	0.002563	0.014291
GLUCOSE1PMETAB-PWY: glucose and glucose-1-phosphate degradation	1.9654	0.002953	0.015712
ENTBACSYN-PWY: enterobactin biosynthesis	2.2311	0.003004	0.015886

PWY-6892: thiazole biosynthesis I (E. coli)	1.9219	0.003513	0.017503
PWY-5910: superpathway of geranylgeranyldiphosphate biosynthesis I (via mevalonate)	-1.4737	0.003864	0.018877
PWY-7269: NAD/NADP-NADH/NADPH mitochondrial interconversion (yeast)	1.8495	0.003942	0.019152
AST-PWY: L-arginine degradation II (AST pathway)	1.5853	0.004115	0.01975
PWY-5265: peptidoglycan biosynthesis II (staphylococci)	-1.2877	0.0042	0.020084
KDO-NAGLIPASYN-PWY: superpathway of (Kdo)2-lipid A biosynthesis	1.6483	0.004882	0.0221
HCAMHPDEG-PWY: 3-phenylpropanoate and 3-(3-hydroxyphenyl)propanoate degradation to 2-oxopent-4-enoate	1.3862	0.004894	0.0221
PWY-6690: cinnamate and 3-hydroxycinnamate degradation to 2-oxopent-4-enoate	1.3862	0.004901	0.0221
PWY-6549: L-glutamine biosynthesis III	-1.2242	0.006627	0.028141
THISYN-PWY: superpathway of thiamin diphosphate biosynthesis I	1.9818	0.007423	0.030839
PWY-5656: mannosylglycerate biosynthesis I	1.1883	0.007839	0.032191
PWY-2723: trehalose degradation V	1.5282	0.00867	0.035257
FUC-RHAMCAT-PWY: superpathway of fucose and rhamnose degradation	1.7256	0.008921	0.035985
PWY-5705: allantoin degradation to glyoxylate III	1.3562	0.010063	0.03908
PWY0-1241: ADP-L-glycero-β-D-manno-heptose biosynthesis	1.5578	0.011113	0.042432
PWY-5083: NAD/NADH phosphorylation and dephosphorylation	1.7207	0.01115	0.04251
PWY-6612: superpathway of tetrahydrofolate biosynthesis	1.61	0.011472	0.043405
FERMENTATION-PWY: mixed acid fermentation	1.6291	0.013317	0.048691

HUMAnN 2.0 pathway feature was utilized to identify relevant pathways between axSpA and HC prior to analysis using EdgeR. Pathways are displayed meeting the criteria of $p<0.05$ and FDR <0.05. Log_2FC displayed, with HC positive and axSpA negative (shaded in gray).

Supplemental Table 6. Relative tryptophan pathway gene abundances in bacterial metagenomics from HC vs. combined axSpA + CD-axSpA groups.

Genes (HC:axSpA+CD-axSpA)	log_2FC	P-value	FDR	Trp Function
K00180: indolepyruvate ferredoxin oxidoreductase, beta subunit Alistipes finegoldii	1.6967	2.19E-05	0.001173	Unclear
K00179: indolepyruvate ferredoxin oxidoreductase, alpha subunit Alistipes finegoldii	1.545	8.82E-05	0.002756	Unclear
K00179: indolepyruvate ferredoxin oxidoreductase, alpha subunit Bacteroides eggerthii	-2.2709	0.000142	0.003612	Unclear
K00180: indolepyruvate ferredoxin oxidoreductase, beta subunit Bacteroides fragilis	1.2715	0.001382	0.015252	Unclear
K00180: indolepyruvate ferredoxin oxidoreductase, beta subunit Parabacteroides distasonis	1.2289	0.001464	0.015869	Unclear
K00180: indolepyruvate ferredoxin oxidoreductase, beta subunit Eggerthella lenta	-1.4414	0.003213	0.027844	Unclear
K00179: indolepyruvate ferredoxin oxidoreductase, alpha subunit Odoribacter splanchnicus	-1.2877	0.006558	0.045641	Unclear
K00180: indolepyruvate ferredoxin oxidoreductase, beta subunit Bacteroides cellulosilyticus	-1.2793	0.00669	0.04629	Unclear
K01609: indole-3-glycerol phosphate synthase Roseburia intestinalis	-2.3638	3.71E-05	0.00164	Synthesis
K01609: indole-3-glycerol phosphate synthase unclassified	-1.9654	0.00147	0.015903	Synthesis
K01609: indole-3-glycerol phosphate synthase Bacteroides thetaiotaomicron	1.2092	0.004571	0.035437	Synthesis
K01609: indole-3-glycerol phosphate synthase Eubacterium rectale	1.2621	0.005467	0.039972	Synthesis
K04103: indolepyruvate decarboxylase	-1.5619	0.000807	0.010674	Metabolis m
K01667: tryptophanase Bacteroides thetaiotaomicron	1.8818	1.24E-06	0.000205	Synthesis
K01667: tryptophanase Alistipes finegoldii	1.5143	0.0001	0.002955	Synthesis
K01667: tryptophanase Porphyromonas asaccharolytica	-2.6805	0.000134	0.003501	Synthesis
K01667: tryptophanase Porphyromonas uenonis	-2.2536	0.00028	0.00539	Synthesis
K07185: tryptophan-rich sensory protein Alistipes finegoldii	1.8952	5.21E-06	0.000485	Signaling
K01867: tryptophanyl-tRNA synthetase Porphyromonas asaccharolytica	-2.9471	4.56E-05	0.001847	Synthesis
K01867: tryptophanyl-tRNA synthetase Bacteroides thetaiotaomicron	1.7	0.000133	0.003479	Synthesis
K01867: tryptophanyl-tRNA synthetase Alistipes finegoldii	1.0745	0.002536	0.023374	Synthesis
K01867: tryptophanyl-tRNA synthetase Ruminococcus bromii	-1.6925	0.002536	0.023374	Synthesis
K01867: tryptophanyl-tRNA synthetase Roseburia intestinalis	-1.4539	0.003362	0.028674	Synthesis
K01867: tryptophanyl-tRNA synthetase Alistipes shahii	-1.3121	0.004075	0.032643	Synthesis
K01695: tryptophan synthase alpha chain Bacteroides thetaiotaomicron	1.2968	0.001056	0.012801	Synthesis
K01695: tryptophan synthase alpha chain Bacteroides fragilis	1.1665	0.003622	0.030085	Synthesis
K01695: tryptophan synthase alpha chain Streptococcus salivarius	1.017	0.005256	0.038895	Synthesis
K01696: tryptophan synthase beta chain Ruminococcus lactaris	1.631	4.92E-05	0.001934	Synthesis
K01696: tryptophan synthase beta chain Roseburia intestinalis	-2.1633	9.60E-05	0.002896	Synthesis
K01696: tryptophan synthase beta chain Bacteroides dorei	1.2728	0.002081	0.020322	Synthesis
K06001: tryptophan synthase beta chain Alistipes shahii	-1.316	0.003218	0.027877	Synthesis
K06001: tryptophan synthase beta chain Roseburia intestinalis	-1.3961	0.003451	0.029178	Synthesis

K01696: tryptophan synthase beta chain Bacteroides cellulosilyticus	-1.3997	0.003495	0.029407	Synthesis
K06001: tryptophan synthase beta chain Alistipes finegoldii	1.0156	0.004599	0.03558	Synthesis
K01696: tryptophan synthase beta chain Bacteroides fragilis	1.1108	0.005576	0.040603	Synthesis
K01695: tryptophan synthase alpha chain Odoribacter splanchnicus	-1.4241	0.001491	0.01537	Synthesis
K01695: tryptophan synthase alpha chain Escherichia coli	-2.0759	0.00299	0.025309	Synthesis
K03835: tryptophan-specific transport protein	-2.0938	0.004385	0.033392	Signaling
K03835: tryptophan-specific transport protein Escherichia coli	-2.0932	0.004916	0.036264	Signaling

KEGG orthology (KO) genes listed with individual genes that were identified as different using EdgeR with $p<0.05$ and FDR <0.05. Genes clustered by same KO. Log_2FC displayed, with HC positive and axSpA-CD-axSpA negative (shaded in gray). Tryptophan function described as either biologically unclear, synthesis, metabolism, or signaling.

Supplemental Table 7. Relative tryptophan pathway gene abundances in bacterial metagenomics from axSpA vs. CD-axSpA groups.

Genes (CD-axSpA:axSpA)	log_2FC	P-value	FDR	Trp Function
K13498: indole-3-glycerol phosphate synthase / phosphoribosylanthranilate isomerase Escherichia coli	3.1243	1.91E-07	1.28E-05	Synthesis
K13498: indole-3-glycerol phosphate synthase / phosphoribosylanthranilate isomerase	3.0405	5.60E-07	2.33E-05	Synthesis
K01609: indole-3-glycerol phosphate synthase Akkermansia muciniphila	3.2742	1.69E-05	0.000203	Synthesis
K01609: indole-3-glycerol phosphate synthase Anaerostipes hadrus	1.7189	0.001813	0.008901	Synthesis
K01609: indole-3-glycerol phosphate synthase Ruminococcus bromii	-2.4763	0.003272	0.014568	Synthesis
K01609: indole-3-glycerol phosphate synthase Odoribacter splanchnicus	1.4248	0.00736	0.029201	Synthesis
K01609: indole-3-glycerol phosphate synthase Parabacteroides distasonis	1.2403	0.007816	0.030723	Synthesis
K04103: indolepyruvate decarboxylase Klebsiella pneumoniae	2.0111	9.64E-05	0.000739	Metabolism
K00179: indolepyruvate ferredoxin oxidoreductase, alpha subunit Bacteroides cellulosilyticus	1.9735	0.000184	0.001247	Unclear
K00180: indolepyruvate ferredoxin oxidoreductase, beta subunit Bacteroides cellulosilyticus	1.9747	0.000489	0.002849	Unclear
K00180: indolepyruvate ferredoxin oxidoreductase, beta subunit Bacteroides caccae	-2.5953	0.002461	0.011473	Unclear
K00179: indolepyruvate ferredoxin oxidoreductase, alpha subunit Bacteroides eggerthii	-2.7507	0.002606	0.012055	Unclear
K00180: indolepyruvate ferredoxin oxidoreductase, beta subunit Eggerthella lenta	1.7739	0.002651	0.012225	Unclear
K00180: indolepyruvate ferredoxin oxidoreductase, beta subunit Bacteroides dorei	-2.465	0.00275	0.012589	Unclear
K00179: indolepyruvate ferredoxin oxidoreductase, alpha subunit Bacteroides dorei	-1.859	0.009778	0.037134	Unclear
K00179: indolepyruvate ferredoxin oxidoreductase, alpha subunit Eggerthella lenta	1.4193	0.011269	0.041701	Unclear
K01867: tryptophanyl-tRNA synthetase Escherichia coli	3.5807	2.24E-07	1.40E-05	Synthesis
K01867: tryptophanyl-tRNA synthetase Clostridium perfringens	2.4126	1.49E-05	0.000185	Synthesis
K01867: tryptophanyl-tRNA synthetase Akkermansia muciniphila	2.7506	5.44E-05	0.000472	Synthesis
K01867: tryptophanyl-tRNA synthetase Porphyromonas asaccharolytica	3.2672	5.98E-05	0.000508	Synthesis
K01867: tryptophanyl-tRNA synthetase Klebsiella pneumoniae	2.0617	7.48E-05	0.000605	Synthesis
K01867: tryptophanyl-tRNA synthetase Clostridium symbiosum	1.626	0.001301	0.006694	Synthesis
K01867: tryptophanyl-tRNA synthetase Bacteroides caccae	-2.7764	0.001527	0.007716	Synthesis
K01867: tryptophanyl-tRNA synthetase Odoribacter splanchnicus	1.6957	0.003037	0.013667	Synthesis
K01867: tryptophanyl-tRNA synthetase Enterococcus faecium	1.3759	0.003837	0.016669	Synthesis
K01867: tryptophanyl-tRNA synthetase Ruminococcus bromii	-2.4093	0.004957	0.02078	Synthesis
K01867: tryptophanyl-tRNA synthetase Eggerthella lenta	1.4126	0.006487	0.02621	Synthesis
K01867: tryptophanyl-tRNA synthetase Porphyromonas uenonis	1.7065	0.007392	0.029294	Synthesis
K01695: tryptophan synthase alpha chain Escherichia coli	3.0304	9.23E-07	3.09E-05	Synthesis

K01695: tryptophan synthase alpha chain Akkermansia muciniphila	2.6029	0.000139	0.000991	Synthesis
K01695: tryptophan synthase alpha chain Bacteroides caccae	-2.2943	0.006513	0.026299	Synthesis
K01695: tryptophan synthase alpha chain Bacteroides dorei	-2.024	0.008762	0.033879	Synthesis
K01695: tryptophan synthase alpha chain Ruminococcus torques	-1.7221	0.010698	0.039892	Synthesis
K01696: tryptophan synthase beta chain Akkermansia muciniphila	3.1155	1.90E-06	4.86E-05	Synthesis
K06001: tryptophan synthase beta chain Akkermansia muciniphila	3.1454	2.84E-06	6.19E-05	Synthesis
K01696: tryptophan synthase beta chain Escherichia coli	2.6536	3.63E-05	0.000348	Synthesis
K01696: tryptophan synthase beta chain Klebsiella pneumoniae	2.0864	6.73E-05	0.000557	Synthesis
K01696: tryptophan synthase beta chain Bacteroides cellulosilyticus	2.1716	0.000142	0.001006	Synthesis
K06001: tryptophan synthase beta chain unclassified	2.252	0.002394	0.011223	Synthesis
K01696: tryptophan synthase beta chain Bacteroides caccae	-2.7	0.002413	0.011282	Synthesis
K06001: tryptophan synthase beta chain Eggerthella lenta	1.4152	0.006416	0.025963	Synthesis
K01696: tryptophan synthase beta chain Ruminococcus bromii	-1.9325	0.013213	0.047461	Synthesis
K03836: low affinity tryptophan permease	2.964	2.11E-06	5.14E-05	Signaling
K01667: tryptophanase Escherichia coli	2.765	7.79E-06	0.000121	Synthesis
K01667: tryptophanase Akkermansia muciniphila	3.0406	1.23E-05	0.000163	Synthesis
K01667: tryptophanase Porphyromonas asaccharolytica	3.3175	3.04E-05	0.000306	Synthesis
K01667: tryptophanase Porphyromonas uenonis	2.7151	0.000175	0.001195	Synthesis
K01667: tryptophanase	2.5719	0.000617	0.003472	Synthesis
K01667: tryptophanase unclassified	2.2237	0.002453	0.011441	Synthesis
K07185: tryptophan-rich sensory protein Clostridium perfringens	2.4318	1.16E-05	0.000156	Signaling
K07185: tryptophan-rich sensory protein	1.7407	0.00519	0.02164	Signaling
K02846: N-methyl-L-tryptophan oxidase	2.489	3.16E-05	0.000315	Synthesis
K02846: N-methyl-L-tryptophan oxidase Escherichia coli	2.489	3.16E-05	0.000315	Synthesis
K03835: tryptophan-specific transport protein Escherichia coli	2.82	5.12E-05	0.000451	Signaling
K03835: tryptophan-specific transport protein	2.7388	0.000103	0.000774	Signaling

KEGG orthology (KO) genes listed with individual genes that were identified as different using EdgeR with p<0.05 and FDR <0.05. Genes clustered by same KO. Log_2FC displayed, with CD-axSpA positive and axSpA negative (shaded in gray). Tryptophan function described as either biologically unclear, synthesis, metabolism, or signaling.

Supplemental Table 8. Relative tryptophan pathway gene abundances in bacterial metagenomics from HC vs CD.

Genes HC:CD	log_2FC	P-value	FDR	Function
K01609: indole-3-glycerol phosphate synthase Akkermansia muciniphila	3.7271	6.68E-08	4.93E-05	Synthesis
K01609: indole-3-glycerol phosphate synthase Campylobacter hominis	2.1774	0.000122	0.002672	Synthesis
K01609: indole-3-glycerol phosphate synthase Odoribacter splanchnicus	-1.6041	0.000595	0.007968	Synthesis
K13498: indole-3-glycerol phosphate synthase / phosphoribosylanthranilate isomerase	-1.9181	0.004107	0.031894	Synthesis
K13498: indole-3-glycerol phosphate synthase / phosphoribosylanthranilate isomerase Escherichia coli	-1.9033	0.004759	0.03531	Synthesis
K00180: indolepyruvate ferredoxin oxidoreductase, beta subunit Odoribacter splanchnicus	-2.0235	6.44E-05	0.001749	Unclear
K00180: indolepyruvate ferredoxin oxidoreductase, beta subunit Bacteroides cellulosilyticus	-1.7158	0.000728	0.009191	Unclear
K00180: indolepyruvate ferredoxin oxidoreductase, beta subunit unclassified	-1.6788	0.00232	0.021019	Unclear
K00180: indolepyruvate ferredoxin oxidoreductase, beta subunit Alistipes shahii	-1.5345	0.002709	0.023559	Unclear
K00179: indolepyruvate ferredoxin oxidoreductase, alpha subunit Odoribacter splanchnicus	-2.0262	7.13E-05	0.001878	Unclear
K00179: indolepyruvate ferredoxin oxidoreductase, alpha subunit Bacteroides cellulosilyticus	-1.4351	0.00528	0.038121	Unclear
K06001: tryptophan synthase beta chain Bacteroides massiliensis	-3.4385	1.19E-06	0.000256	Synthesis
K01696: tryptophan synthase beta chain Bacteroides massiliensis	-3.3616	1.29E-06	0.00027	Synthesis
K01696: tryptophan synthase beta chain Akkermansia muciniphila	2.6064	1.37E-05	0.000676	Synthesis
K06001: tryptophan synthase beta chain Odoribacter splanchnicus	-2.1578	2.95E-05	0.001072	Synthesis
K06001: tryptophan synthase beta chain Akkermansia muciniphila	2.3875	3.85E-05	0.00126	Synthesis
K01696: tryptophan synthase beta chain Campylobacter hominis	1.9963	0.000579	0.007816	Synthesis
K01696: tryptophan synthase beta chain Odoribacter splanchnicus	-1.576	0.0007	0.008926	Synthesis
K01696: tryptophan synthase beta chain Bacteroides cellulosilyticus	-1.7252	0.000786	0.009699	Synthesis
K06001: tryptophan synthase beta chain Alistipes shahii	-1.3056	0.003811	0.030139	Synthesis
K01696: tryptophan synthase beta chain Haemophilus parainfluenzae	-1.3415	0.004143	0.032111	Synthesis
K06001: tryptophan synthase beta chain Bacteroides dorei	-1.8956	0.004328	0.033078	Synthesis
K03836: low affinity tryptophan permease	-3.1777	4.29E-06	0.000417	Signaling
K03836: low affinity tryptophan permease Escherichia coli	-3.1777	4.29E-06	0.000417	Signaling
K01867: tryptophanyl-tRNA synthetase Bacteroides massiliensis	-3.2003	7.76E-06	0.000505	Synthesis
K01867: tryptophanyl-tRNA synthetase Akkermansia muciniphila	2.5117	2.81E-05	0.001038	Synthesis
K01867: tryptophanyl-tRNA synthetase Campylobacter hominis	2.1081	0.000259	0.004418	Synthesis
K01867: tryptophanyl-tRNA synthetase Odoribacter splanchnicus	-1.5328	0.000826	0.010049	Synthesis
K01867: tryptophanyl-tRNA synthetase Bacteroides dorei	-2.1673	0.001425	0.014887	Synthesis
K01867: tryptophanyl-tRNA synthetase Alistipes shahii	-1.5387	0.001901	0.018219	Synthesis
K01867: tryptophanyl-tRNA synthetase Barnesiella intestinihominis	-1.4381	0.003455	0.028106	Synthesis
K01667: tryptophanase Escherichia coli	-2.6953	3.42E-05	0.001175	Synthesis
K01667: tryptophanase Odoribacter splanchnicus	-1.6095	0.000443	0.006485	Synthesis
K01667: tryptophanase Akkermansia muciniphila	1.8314	0.001358	0.014423	Synthesis
K01695: tryptophan synthase alpha chain Campylobacter hominis	1.7378	0.001346	0.014337	Synthesis

KEGG orthology (KO) genes listed with individual genes that were identified as different using EdgeR with $p<0.05$ and FDR <0.05. Genes clustered by same KO. Log_2FC displayed, with HC positive and CD negative (shaded in gray). Tryptophan function described as either biologically unclear, synthesis, metabolism, or signaling.

Supplemental Table 9. Relative tryptophan pathway gene abundances in bacterial metagenomics from CD-axSpA vs HC groups.

Genes (CD-axSpA:HC)	log_2FC	P-value	FDR	Trp Function
K01609: indole-3-glycerol phosphate synthase Roseburia intestinalis	2.0781	8.88E-05	0.001421	Synthesis
K01609: indole-3-glycerol phosphate synthase Bacteroides thetaiotaomicron	-1.8727	0.00605	0.031784	Synthesis
K01609: indole-3-glycerol phosphate synthase Bacteroides dorei	-1.856	0.007745	0.038214	Synthesis
K04103: indolepyruvate decarboxylase	2.0015	0.000117	0.001723	Metabolism
K04103: indolepyruvate decarboxylase Klebsiella pneumoniae	2.0015	0.000117	0.001723	Metabolism
K00179: indolepyruvate ferredoxin oxidoreductase, alpha subunit Bacteroides cellulosilyticus	1.7831	0.001249	0.009766	Unclear
K00179: indolepyruvate ferredoxin oxidoreductase, alpha subunit Bacteroides dorei	-2.1822	0.002752	0.017692	Unclear
K00180: indolepyruvate ferredoxin oxidoreductase, beta subunit Bacteroides caccae	-2.372	0.002772	0.017779	Unclear
K00180: indolepyruvate ferredoxin oxidoreductase, beta subunit Bacteroides dorei	-2.2409	0.003748	0.022124	Unclear
K01867: tryptophanyl-tRNA synthetase Porphyromonas asaccharolytica	4.2595	1.06E-07	2.24E-05	Synthesis
K01867: tryptophanyl-tRNA synthetase Clostridium perfringens	2.2512	7.92E-05	0.001316	Synthesis
K01867: tryptophanyl-tRNA synthetase Klebsiella pneumoniae	2.0525	9.19E-05	0.001456	Synthesis
K01867: tryptophanyl-tRNA synthetase Clostridium symbiosum	1.8121	0.000316	0.003502	Synthesis
K01867: tryptophanyl-tRNA synthetase unclassified	2.4231	0.000406	0.004284	Synthesis
K01867: tryptophanyl-tRNA synthetase Porphyromonas uenonis	2.1746	0.000552	0.00534	Synthesis
K01867: tryptophanyl-tRNA synthetase Bacteroides caccae	-2.2635	0.002637	0.01713	Synthesis
K01867: tryptophanyl-tRNA synthetase Alistipes shahii	1.4581	0.005272	0.028709	Synthesis
K01867: tryptophanyl-tRNA synthetase Enterococcus faecium	1.3728	0.005999	0.031585	Synthesis
K01867: tryptophanyl-tRNA synthetase Anaerostipes hadrus	1.329	0.007102	0.035804	Synthesis
K01867: tryptophanyl-tRNA synthetase Bacteroides thetaiotaomicron	-1.9581	0.010125	0.04645	Synthesis
K01667: tryptophanase Porphyromonas asaccharolytica	4.002	3.15E-07	3.51E-05	Synthesis
K01667: tryptophanase Porphyromonas uenonis	3.4521	1.01E-06	6.20E-05	Synthesis
K01667: tryptophanase Escherichia coli	2.3153	0.00024	0.002886	Synthesis
K01667: tryptophanase unclassified	2.4576	0.000514	0.005057	Synthesis
K01667: tryptophanase	2.2788	0.002461	0.016288	Synthesis
K01667: tryptophanase Alistipes shahii	1.5905	0.002574	0.016801	Synthesis
K01667: tryptophanase Bacteroides thetaiotaomicron	-1.7545	0.008442	0.040729	Synthesis
K01696: tryptophan synthase beta chain Roseburia intestinalis	2.4427	1.35E-05	0.000359	Synthesis
K01696: tryptophan synthase beta chain Bacteroides cellulosilyticus	2.4494	1.90E-05	0.000463	Synthesis
K01696: tryptophan synthase beta chain Klebsiella pneumoniae	2.0773	8.31E-05	0.001364	Synthesis
K06001: tryptophan synthase beta chain unclassified	2.5158	0.000578	0.00552	Synthesis
K06001: tryptophan synthase beta chain Alistipes shahii	1.752	0.001022	0.008397	Synthesis
K01696: tryptophan synthase beta chain Bacteroides caccae	-2.2829	0.00352	0.021196	Synthesis
K01696: tryptophan synthase beta chain Bacteroides dorei	-1.8651	0.006774	0.034557	Synthesis
K06001: tryptophan synthase beta chain Roseburia intestinalis	1.3221	0.007061	0.03565	Synthesis
K03836: low affinity tryptophan permease	2.4854	0.000113	0.001679	Signaling
K03836: low affinity tryptophan permease Escherichia coli	2.4854	0.000113	0.001679	Signaling
K07185: tryptophan-rich sensory protein Clostridium perfringens	1.9694	0.000691	0.006284	Signaling

K07185: tryptophan-rich sensory protein Alistipes finegoldii	-1.8776	0.009588	0.044607	Signaling
K01695: tryptophan synthase alpha chain Klebsiella pneumoniae	1.5227	0.00188	0.013382	Synthesis
K01695: tryptophan synthase alpha chain Bacteroides dorei	-1.9163	0.006784	0.034577	Synthesis

KEGG orthology (KO) genes listed with individual genes that were identified as different using EdgeR with $p<0.05$ and FDR <0.05. Genes clustered by same KO. Log_2FC displayed, with CD-axSpA positive and HC negative (shaded in gray). Tryptophan function described as either biologically unclear, synthesis, metabolism, or signaling.

Supplemental Table 10. Relative tryptophan pathway gene abundances in bacterial metagenomics from axSpA vs. CD-axSpA groups.

Genes (CD-axSpA:CD)	log_2FC	P-value	FDR	Trp Function
K01609: indole-3-glycerol phosphate synthase Akkermansia muciniphila	4.8458	3.39E-12	8.24E-09	Synthesis
K01609: indole-3-glycerol phosphate synthase Anaerostipes hadrus	2.2496	1.32E-05	0.000334	Synthesis
K01609: indole-3-glycerol phosphate synthase Roseburia intestinalis	1.8746	0.000304	0.003217	Synthesis
K01609: indole-3-glycerol phosphate synthase Campylobacter hominis	2.1504	0.000548	0.004724	Synthesis
K01609: indole-3-glycerol phosphate synthase Bacteroides dorei	-3.1691	0.000827	0.006185	Synthesis
K01609: indole-3-glycerol phosphate synthase Ruminococcus torques	-2.4718	0.001219	0.008015	Synthesis
K01609: indole-3-glycerol phosphate synthase Bacteroides thetaiotaomicron	-2.2113	0.004509	0.021072	Synthesis
K01609: indole-3-glycerol phosphate synthase Streptococcus salivarius	0.9201	0.024366	0.0863	Synthesis
K01609: indole-3-glycerol phosphate synthase Bacteroides massiliensis	-1.1389	0.041032	0.13175	Synthesis
K01609: indole-3-glycerol phosphate synthase Eubacterium eligens	0.79786	0.080217	0.22119	Synthesis
K01609: indole-3-glycerol phosphate synthase Lachnospiraceae_bacterium_5_1_63FAA	0.78473	0.14676	0.34817	Synthesis
K00180: indolepyruvate ferredoxin oxidoreductase, beta subunit Eggerthella lenta	2.1733	9.41E-05	0.001444	Unclear
K00180: indolepyruvate ferredoxin oxidoreductase, beta subunit Bacteroides caccae	-3.2106	0.000354	0.00357	Unclear
K00180: indolepyruvate ferredoxin oxidoreductase, beta subunit Bacteroides dorei	-3.2587	0.000913	0.006581	Unclear
K00180: indolepyruvate ferredoxin oxidoreductase, beta subunit unclassified	-2.2255	0.005412	0.024416	Unclear
K00180: indolepyruvate ferredoxin oxidoreductase, beta subunit Bacteroides thetaiotaomicron	-2.0377	0.011376	0.045356	Unclear
K00180: indolepyruvate ferredoxin oxidoreductase, beta subunit Alistipes finegoldii	-1.6522	0.017185	0.064585	Unclear
K00180: indolepyruvate ferredoxin oxidoreductase, beta subunit Eubacterium siraeum	1.0663	0.021527	0.077818	Unclear
K00180: indolepyruvate ferredoxin oxidoreductase, beta subunit Bacteroides finegoldii	-1.0785	0.059418	0.17492	Unclear
K00180: indolepyruvate ferredoxin oxidoreductase, beta subunit Eubacterium eligens	0.96198	0.062351	0.18202	Unclear
K00180: indolepyruvate ferredoxin oxidoreductase, beta subunit Bacteroides faecis	-0.9936	0.10305	0.26731	Unclear
K00180: indolepyruvate ferredoxin oxidoreductase, beta subunit Parabacteroides distasonis	-0.83756	0.13789	0.33268	Unclear
K00180: indolepyruvate ferredoxin oxidoreductase, beta subunit	-1.2476	0.16619	0.38096	Unclear
K00180: indolepyruvate ferredoxin oxidoreductase, beta subunit Bacteroides massiliensis	-0.67685	0.20355	0.43999	Unclear
K00180: indolepyruvate ferredoxin oxidoreductase, beta subunit Alistipes shahii	-0.84197	0.24077	0.49633	Unclear
K00179: indolepyruvate ferredoxin oxidoreductase, alpha subunit Eggerthella lenta	1.7719	0.000526	0.004587	Unclear
K00179: indolepyruvate ferredoxin oxidoreductase, alpha subunit Bacteroides dorei	-3.4199	0.00056	0.004788	Unclear
K00179: indolepyruvate ferredoxin oxidoreductase, alpha subunit Alistipes finegoldii	-1.6876	0.017164	0.064521	Unclear

K00179: indolepyruvate ferredoxin oxidoreductase, alpha subunit Bacteroides uniformis	-1.6756	0.038793	0.12626	Unclear
K00179: indolepyruvate ferredoxin oxidoreductase, alpha subunit Bacteroides stercoris	-1.4897	0.048937	0.15091	Unclear
K00179: indolepyruvate ferredoxin oxidoreductase, alpha subunit Eubacterium siraeum	0.77135	0.087537	0.23607	Unclear
K00179: indolepyruvate ferredoxin oxidoreductase, alpha subunit unclassified	1.0065	0.095497	0.25164	Unclear
K00179: indolepyruvate ferredoxin oxidoreductase, alpha subunit Eubacterium eligens	0.75222	0.10593	0.27291	Unclear
K04103: indolepyruvate decarboxylase Klebsiella pneumoniae	1.7346	0.00065	0.005265	Metabolism
K04103: indolepyruvate decarboxylase	1.5784	0.001972	0.011228	Metabolism
K06001: tryptophan synthase beta chain Akkermansia muciniphila	3.6762	4.93E-09	5.51E-07	Synthesis
K01696: tryptophan synthase beta chain Akkermansia muciniphila	3.5614	6.02E-09	6.28E-07	Synthesis
K06001: tryptophan synthase beta chain Bacteroides massiliensis	-3.8777	0.00017	0.002196	Synthesis
K01696: tryptophan synthase beta chain Anaerostipes hadrus	1.7486	0.000267	0.002954	Synthesis
K01696: tryptophan synthase beta chain Klebsiella pneumoniae	1.8522	0.000296	0.003166	Synthesis
K01696: tryptophan synthase beta chain Bacteroides caccae	-3.08	0.000629	0.005153	Synthesis
K01696: tryptophan synthase beta chain Bacteroides massiliensis	-3.3975	0.000647	0.005252	Synthesis
K01696: tryptophan synthase beta chain Roseburia intestinalis	1.913	0.000712	0.005594	Synthesis
K06001: tryptophan synthase beta chain Bacteroides dorei	-3.282	0.0008	0.006041	Synthesis
K01696: tryptophan synthase beta chain Bacteroides dorei	-3.1655	0.001027	0.007141	Synthesis
K06001: tryptophan synthase beta chain Roseburia intestinalis	1.489	0.001495	0.009218	Synthesis
K01696: tryptophan synthase beta chain Ruminococcus torques	-2.31	0.002969	0.015206	Synthesis
K01696: tryptophan synthase beta chain Bacteroides thetaiotaomicron	-2.4063	0.003684	0.01794	Synthesis
K06001: tryptophan synthase beta chain Eggerthella lenta	1.4282	0.004535	0.021161	Synthesis
K01696: tryptophan synthase beta chain Campylobacter hominis	1.675	0.006219	0.027322	Synthesis
K01695: tryptophan synthase alpha chain Akkermansia muciniphila	3.649	5.13E-09	5.53E-07	Synthesis
K01695: tryptophan synthase alpha chain Klebsiella pneumoniae	1.5294	0.001131	0.007627	Synthesis
K01695: tryptophan synthase alpha chain Bacteroides dorei	-2.9105	0.001631	0.009771	Synthesis
K01695: tryptophan synthase alpha chain Bacteroides caccae	-2.5741	0.00186	0.010738	Synthesis
K01695: tryptophan synthase alpha chain Anaerostipes hadrus	1.4389	0.002202	0.0122	Synthesis
K01695: tryptophan synthase alpha chain Campylobacter hominis	1.7786	0.003166	0.015976	Synthesis
K01695: tryptophan synthase alpha chain Ruminococcus torques	-2.2227	0.003879	0.018689	Synthesis
K01695: tryptophan synthase alpha chain Lachnospiraceae bacterium 5_1_63FAA	1.6424	0.004155	0.019762	Synthesis
K01695: tryptophan synthase alpha chain Bacteroides thetaiotaomicron	-2.0377	0.008937	0.036991	Synthesis
K01867: tryptophanyl-tRNA synthetase Akkermansia muciniphila	3.5741	7.96E-09	7.26E-07	Synthesis
K01867: tryptophanyl-tRNA synthetase Clostridium perfringens	2.4079	7.67E-06	0.000218	Synthesis
K01867: tryptophanyl-tRNA synthetase Klebsiella pneumoniae	2.0585	3.85E-05	0.000761	Synthesis
K01867: tryptophanyl-tRNA synthetase Bacteroides massiliensis	-4.0443	0.000141	0.001927	Synthesis
K01867: tryptophanyl-tRNA synthetase Porphyromonas asaccharolytica	3.0037	0.000186	0.002319	Synthesis
K01867: tryptophanyl-tRNA synthetase Clostridium symbiosum	1.7256	0.00034	0.003475	Synthesis
K01867: tryptophanyl-tRNA synthetase Bacteroides caccae	-3.3342	0.000358	0.003596	Synthesis
K01867: tryptophanyl-tRNA synthetase Bacteroides dorei	-3.4283	0.000662	0.005334	Synthesis
K01867: tryptophanyl-tRNA synthetase Campylobacter hominis	1.9089	0.00215	0.011992	Synthesis
K01867: tryptophanyl-tRNA synthetase Anaerostipes hadrus	1.4406	0.002375	0.01285	Synthesis

K01867: tryptophanyl-tRNA synthetase Roseburia intestinalis	1.3601	0.002651	0.013971	Synthesis
K01867: tryptophanyl-tRNA synthetase Porphyromonas uenonis	1.8552	0.002894	0.014907	Synthesis
K01867: tryptophanyl-tRNA synthetase Eggerthella lenta	1.4719	0.003266	0.016331	Synthesis
K01867: tryptophanyl-tRNA synthetase Bacteroides thetaiotaomicron	- 2.3302	0.005279	0.023921	Synthesis
K01667: tryptophanase Akkermansia muciniphila	3.4108	1.38E-07	6.93E-06	Synthesis
K01667: tryptophanase Porphyromonas uenonis	3.0891	9.16E-06	0.000252	Synthesis
K01667: tryptophanase Porphyromonas asaccharolytica	3.1159	5.83E-05	0.001033	Synthesis
K01667: tryptophanase unclassified	2.4394	0.000523	0.004573	Synthesis
K01667: tryptophanase Bacteroides thetaiotaomicron	-2.1527	0.00737	0.031465	Synthesis
K07185: tryptophan-rich sensory protein Clostridium perfringens	2.4269	5.79E-06	0.000173	Signaling

KEGG orthology (KO) genes listed with individual genes that were identified as different using EdgeR with $p<0.05$ and FDR <0.05. Genes clustered by same KO. Log_2FC displayed, with CD-axSpA positive and CD negative (shaded in gray). Tryptophan function described as either biologically unclear, synthesis, metabolism, or signaling.
